# Compressive stress–driven Piezo1 activation and Rho-ROCK mechanotransduction promote tumor progression via epigenetic mechanical memory

**DOI:** 10.1126/sciadv.aeb1271

**Published:** 2026-03-04

**Authors:** Sarah T. Boyle, David Gallego-Ortega, Edward J. Buckley, Emmanuelle Cognard, M. Zahied Johan, Zahra Esmaeili, Makoto Kamei, Kate Poole, Michael S. Samuel

**Affiliations:** ^1^ Centre for Cancer Biology, SA Pathology and Adelaide University, Adelaide, SA 5000, Australia.; ^2^ University of Technology Sydney, Ultimo, NSW 2007, Australia.; ^3^ University of New South Wales, Sydney, NSW 2052, Australia.; ^4^ Garvan Institute of Medical Research, Sydney, NSW 2010, Australia.; ^5^ Basil Hetzel Institute for Translational Health Research, Woodville South, SA 5011, Australia.; ^6^ School of Biomedical Sciences, Faculty of Medicine and Health, University of New South Wales, Sydney, NSW 2052, Australia.

## Abstract

Rapidly growing tumors experience high tissue-level forces, particularly when growing within a restricted space. These require counteracting by intracellular forces to prevent tissue damage. Here, we reveal the ion channel Piezo1 as a mechanosensor of compressive force, activating Rho–Rho kinase (ROCK) mechanotransduction to generate intracellular forces and enhancing malignant characteristics of tumors. Compressive stress promoted cancer growth in vivo in a Rho-ROCK–dependent manner. Silencing Piezo1 abolished compression-induced Rho-ROCK activation and tumor progression in this model. Accordingly, elevated PIEZO1 is associated with 35% poorer survival of patients with breast cancer. We show that acute compressive forces engender epigenetic mechanical memory via Piezo1-activated Rho-ROCK signaling, promoting tumor growth in vivo. Compressive stress promoted ROCK-dependent histone modifications associated with open chromatin, including acetylation of key histone 3–lysine residues, regulating the expression of cancer-related genes across cell, explant, and in vivo tumor models. Our observations suggest that the PIEZO1-RHO-ROCK axis links tissue-level forces to persistent tumor-promoting epigenetic changes and merits evaluation as a mechanotherapy target in cancer.

## Teaser

Compressive force drives PIEZO1-RHO-ROCK mechanotransduction and establishes an epigenetic memory that promotes tumor progression.

## INTRODUCTION

In embryonic development and adult life, normal cells and tissues are subjected to diverse mechanical inputs including tensile force, shear force, and compressive force that trigger intracellular mechanotransduction signaling pathways. These forces contribute to the maintenance of cell shape, division, differentiation, and organ/tissue formation in a temporally and spatially regulated manner. However, conditions such as edema or tumor growth may generate pathologically heightened external forces that must be balanced intracellularly via a process referred to as mechanoreciprocity, which regulates cellular responses (*1*).

The influence of compressive forces on the biology of load-bearing tissues such as bones, periodontal tissues, cartilage tissue, and muscle is widely recognized, and precisely targeted compressive force is similarly well established as essential for accurate tissue folding in organ development (*2*). For example, each stage of mammary gland development, including embryonic, pubertal, pregnancy, lactation, and involution, is exquisitely influenced by unique mechanical inputs (*3*). On the other hand, during growth of a solid cancer, cells within the tumor are subjected to high levels of dynamic compressive force exerted by the compact tumor core, neighboring cancer and stromal cells, confinement by the extracellular matrix (ECM) and ECM capsules formed by cancer-associated fibroblasts (*4*), and/or rapid proliferation within a restricted space, such as within the mammary duct (*3*, *5*, *6*). Hence, it is expected that dysregulation of mechanoreciprocity is associated with cancer progression (*7*–*9*), including in the breast (*3*).

We previously demonstrated that the application of acute compressive stress to a model cell line [human embryonic kidney (HEK) 293T] and native, intact epithelial tissues, including the mouse mammary gland, activated RhoA–Rho kinase (ROCK) signaling. Compressive stress elevated active RhoA–guanosine 5′-triphosphate (GTP) and downstream phosphorylation of the ROCK substrate myosin light chain–2 (MLC2) levels within 30 min, leading to important changes in cell phenotype (*10*). We therefore sought to understand the role of compressive mechanical stress in tumor growth and progression in breast cancer, where incipient tumor growth occurs within the constriction of the mammary lactiferous ducts.

While the role of integrins in sensing changes in ECM stiffness to activate Rho-ROCK signaling has been well canvassed (*11*), the identity of protein(s) that sense and respond to compressive force to induce compensatory intracellular forces has remained elusive. Mechanosensitive ion channels (MSICs) are well conserved throughout evolution and can be activated in response to mechanical forces. They play key roles in the cellular response to externally applied force (*12*). Eukaryotes have DEG/ENaC, transient receptor potential (TRP), and PIEZO classes of MSICs (*13*). Here, we investigated whether MSICs have a role in mechanosensing compressive stress, revealing a specific role for Piezo1 in this process. Under compressive stress, Piezo1 permits rapid Ca^2+^ ion influx resulting in calcium- and calmodulin-dependent protein kinase II (CaMKII)–driven RhoA-ROCK signaling in intact mammary tumors and isolated cancer cells maintained within three-dimensional (3D) matrices. Compressive stress–induced intracellular force markedly alters cell functionality and fate, including increased proliferation, transition to a mesenchymal phenotype, and enhanced tumor growth in vivo. Long-lived cellular consequences are associated with substantial histone modifications and enhanced gene activation, suggesting that acute compressive force generates Piezo1-Ca^2+^-CaMKII-RhoA-ROCK–dependent mechanical memory that promotes tumor progression.

## RESULTS

### Compressive stress promotes mammary cancer growth and activates the Rho-ROCK mechanotransduction signaling pathway

**Fig. 1.**
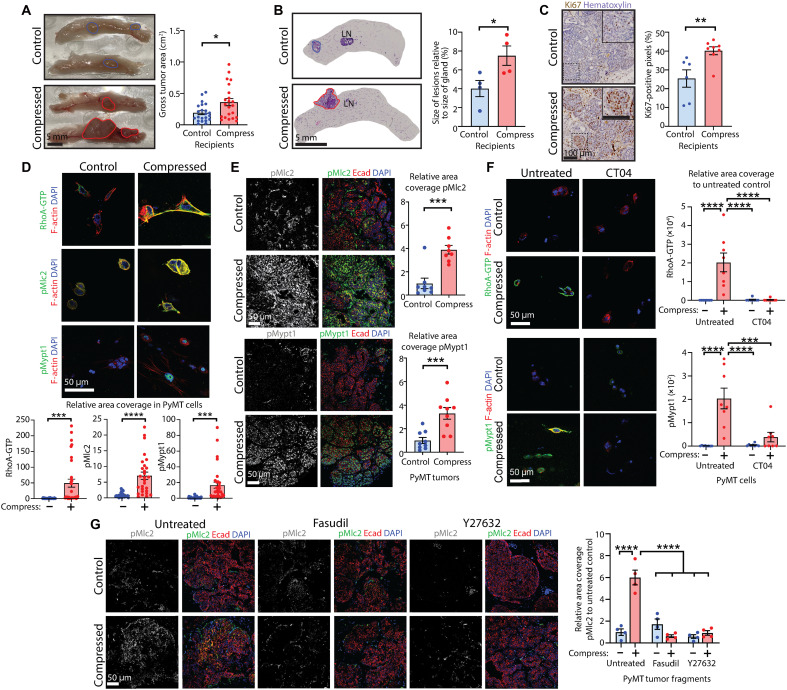
Compressive stress promotes mammary cancer growth and activates the Rho-ROCK mechanotransduction signaling pathway. (**A**) Tumors formed from transplantation of tumor tissue fragments subjected to compression. Scale bar, 5 mm. Chart: tumor area; mean ± SEM (*n* = 22 glands per group; three pooled experiments); unpaired *t* test. (**B**) Tumors formed by transplantation of tumor tissue fragments subjected to compression, stained with hematoxylin and eosin. Scale bar, 5 mm. Chart: percentage of gland containing lesions; mean ± SEM (*n* = 4 glands per group); unpaired *t* test. LN, Lymph node. (**C**) Ki67 immunohistochemistry of tumors from (A) and (B), counterstained with hematoxylin. Scale bars, 100 μm. Insets show magnification. Chart: percentage of positive pixels; mean ± SEM (*n* = 6 control and 8 compressed); unpaired *t* test. (**D**) RhoA-GTP, phospho-Mlc2 (pMlc2), and phospho–myosin phosphatase target subunit-1 (pMypt1) quantitative immunofluorescence in tumor cells subjected to compression in collagen matrices, also labeled for filamentous (F)–actin and 4′,6-diamidino-2-phenylindole (DAPI). Scale bar, 50 μm. Charts: relative area coverage of signal; mean ± SEM [RhoA-GTP *n* = 31 control and 32 compressed fields of view (FOV), pMlc2 *n* = 27 per group, and pMypt1 *n* = 30 per group; four pooled experiments]; unpaired *t* tests. (**E**) pMlc2 and pMypt quantitative immunofluorescence in compressed tumor tissue, also labeled for E-cadherin (Ecad) and DAPI. Scale bars, 50 μm. Charts: relative area coverage; mean ± SEM (*n* = 9 tumors per group); unpaired *t* tests. (**F**) RhoA-GTP and pMypt1 in tumor cells subjected to compression in collagen, pretreated with Rho inhibitor CT04, also labeled for F-actin and DAPI. Scale bars, 50 μm. Charts: relative area coverage; mean ± SEM (*n* = 8 FOV per group), analysis of variance (ANOVA). (**G**) pMlc2 in tumor tissue pretreated with ROCK inhibitor Fasudil or Y27632 before compression, also labeled for E-cadherin and DAPI. Scale bar, 50 μm. Chart: relative area coverage; mean ± SEM (*n* = 4 tumor fragments per group); ANOVA. *P < 0.05, **P < 0.01, ***P < 0.001, and ****P < 0.0001.

To first establish whether compressive force promotes mammary cancer growth in vivo, we used the murine mammary tumor virus–driven polyomavirus middle T antigen (MMTV-PyMT) transgenic mouse model of breast cancer (*14*) coupled with a congenic tissue transplantation approach. Early-stage primary mammary tumors from PyMT mice (7 to 8 weeks old) were subjected to acute compressive stress for 30 min at 20 kPa using the Flexcell FX-5000 Compression System (see schematic fig. S1A) as we have previously optimized (*10*), and 1-mm^3^ fragments were transplanted orthotopically into the mammary fat pads of congenic FVB/n wild-type (WT) recipients. After 4 weeks, glands were dissected out, and the extent of tumorigenesis evaluated macroscopically and histologically. We found that the tumor area (Fig. 1A) and percentage of the mammary fat pads encompassed by tumorigenic lesions (Fig. 1B) were significantly higher in recipient glands containing compressed tumor tissue compared to those containing uncompressed control tumor tissue. Furthermore, tumors formed from tissue subjected to compressive stress exhibited more proliferation compared to uncompressed controls, as assessed by Antigen Kiel 67 (Ki67) immunolabeling (Fig. 1C), without any alterations in apoptosis levels (fig. S1B). These data suggest that acute compressive stress enhances tumor cell proliferation and tumor growth.

We previously showed that activation of the Rho-ROCK signaling pathway could be induced by the application of compressive force (*10*), consistent with other studies in cartilage and chondrocytes that are subjected to heavy mechanical loads (*15*, *16*). When tissue from transplant donors was assessed, we found enhanced phosphorylation of the ROCK substrate Mlc2 at Ser^19^ (*17*) in compressed samples, indicating enhanced ROCK activity before transplantation (fig. S1C). We therefore investigated activation of mechanotransduction pathways downstream of compressive stress, using primary PyMT mammary tumor cells embedded in a collagen matrix as a physiologically relevant biomaterial (see schematic fig. S1D) or using whole PyMT tumors that were fixed immediately following compression (fig. S1A). RhoA-ROCK signaling was robustly activated by compressive stress, as shown by higher levels of active GTP-bound RhoA, phospho-Mlc2 (pMlc2), and phosphorylation at Thr^696^ of another ROCK substrate, myosin phosphatase target subunit-1 (Mypt1) (*18*) in tumor cells in collagen matrices (Fig. 1D), and higher pMlc2 and phospho-Mypt1 (pMypt1) in tumors (Fig. 1E). RhoA was active within 5 min of compression, with activation of the entire pathway within 30 min of compression (fig. S1E), mirroring our previous findings (*10*). Supporting this, immunofluorescence analysis of PyMT primary tumor cells treated ex vivo with the Rho activator CN03 for 30 min also exhibited Rho-ROCK pathway activation as evidenced by elevated RhoA-GTP and phosphorylation of Mlc2 and Mypt1 (fig. S1F).

Investigating signaling through other established mechanotransduction pathways, we observed low-level activation of focal adhesion kinase (FAK; Tyr^397^-phosphorylated FAK) in tumor cells and whole tumors (fig. S1, G and H) upon compression, although no downstream activation of phosphatidylinositol 3-kinase (PI3K)/Akt signaling was observed (assessed via Ser^473^ phosphorylation of Akt; fig. S1I). Furthermore, application of compressive stress did not affect glycogen synthase kinase (GSK) 3β activity (assessed via Ser^9^ phosphorylation; fig. S1J), stabilization or activation of β-catenin (fig. S1K), activation of Hippo signaling [active (nonphosphorylated) YAP1; fig. S1L], or enhance signaling through the nuclear factor κB (NF-κB) pathway (by assessing phosphorylation of RELA/NF-κB p65 at Ser^536^; fig. S1M). To substantiate the finding that compressive stress enhances signaling through ROCK via activation of RhoA in mammary cancer cells, we pretreated PyMT cells embedded in collagen with Rho inhibitor I (CT04), a cell-permeable version of exoenzyme C3 transferase from *Clostridium botulinum*, to inactivate Rho before compression (see schematic fig. S1D). Inhibition of Rho blocked both compressive stress–induced activation of RhoA and downstream ROCK activity (Fig. 1F). Furthermore, pretreatment of intact tumor fragments with Fasudil or Y27632, structurally unrelated inhibitors of ROCK kinase activity, blocked ROCK-mediated phosphorylation of Mlc2 downstream of compressive stress (Fig. 1G). These data show that the application of compressive stress selectively activates mechanotransduction signaling through the RhoA-ROCK pathway in primary mammary tumor tissue and cells. It does not activate other mechanotransduction pathways such as those mediated by β-catenin, PI3K/Akt, YAP, or NF-κB.

### Compressive force is sensed by the MSIC Piezo1

**Fig. 2.**
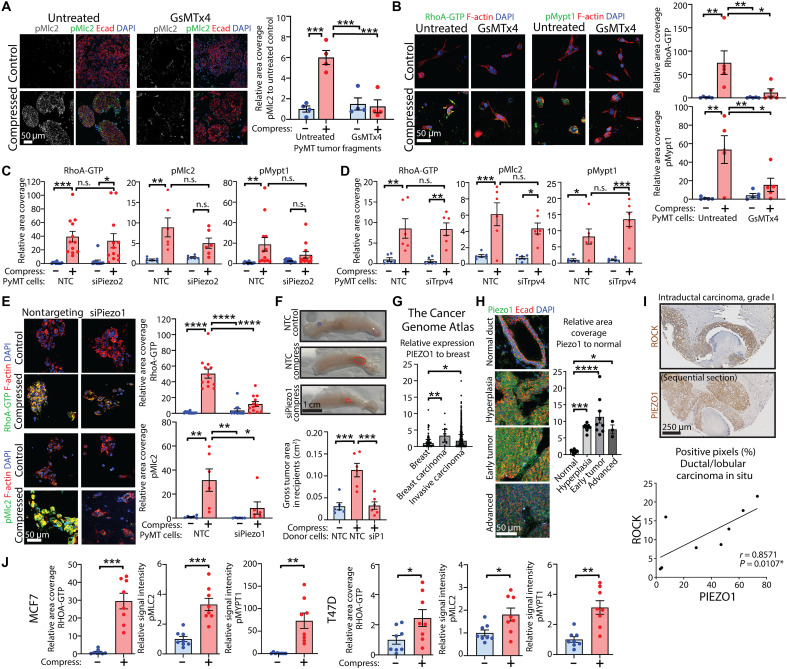
Compressive stress is sensed by the MSIC Piezo1. (**A**) pMlc2 in tumor tissue pretreated with GsMTx4 and compressed, colabeled for E-cadherin. Scale bar, 50 μm. Chart: relative area coverage; mean ± SEM (*n* = 4 tumor fragments per group); ANOVA. (**B**) RhoA-GTP and pMypt1 in tumor cells treated with GsMTx4 and compressed, colabeled for F-actin. Scale bar, 50 μm. Charts: relative area coverage; mean ± SEM (*n* = 5 FOV per group); ANOVA. (**C** and **D**) RhoA-GTP, pMlc2, and pMypt1 quantitative immunofluorescence in tumor cells ± *Piezo2* (C) or *Trpv4* (D) knockdown and compression. Charts: mean ± SEM [(C): *n* = 12 FOV per group RhoA-GTP/pMypt1 and *n* = 6 pMlc2; (D): *n* = 6 per group]; ANOVA. n.s., not significant. (**E**) RhoA-GTP and pMlc2 in tumor cells ± *Piezo1* knockdown and compression, colabeled for F-actin. Scale bar, 50 μm. Charts: relative area coverage; mean ± SEM (*n* = 12 FOV per group for RhoA-GTP and 6 per group for pMlc2); ANOVA. (**F**) Tumors formed from transplantation of compressed cells ± *Piezo1* knockdown. Scale bar, 1 cm. Chart: tumor area; mean ± SEM (*n* = 6 per group); ANOVA. SiP1, siPiezo1. (**G**) Relative *PIEZO1* mRNA in human breast or breast cancer samples; The Cancer Genome Atlas (TCGA). Median ± interquartile range (*n* = 61 breast, 17 carcinoma, and 504 invasive carcinoma); Kruskal-Wallis. (**H**) Piezo1 immunofluorescence in normal mouse mammary gland and tissue taken from PyMT transgenic mice at different stages of tumor development, colabeled for E-cadherin. Scale bar, 50 μm. Chart: area coverage relative to normal duct; mean ± SEM (*n* = 10 normal, 8 hyperplasia, 9 early, and 3 advanced); ANOVA. (**I**) Immunohistochemistry of PIEZO1 and ROCK in human intraductal carcinoma on a breast cancer TMA, counterstained with hematoxylin. Scale bar, 250 μm. Graph: PIEZO1 versus ROCK-positive pixel analysis in ductal and lobular carcinoma in situ samples (*n* = 8), with Spearman correlation coefficient, two-sided test. (**J**) Quantification of immunofluorescence (images in fig. S3G) of RHOA-GTP, pMLC2, and pMYPT1 in MCF7 and T47D human breast cancer cells subjected to compression in collagen matrices. Charts: relative area coverage or intensity of signal; mean ± SEM (*n* = 8 FOV per group); unpaired *t* tests. *P < 0.05, **P < 0.01, ***P < 0.001, and ****P < 0.0001.

While it is known that Rho-ROCK signaling can be activated downstream of a stiffer ECM via integrin-mediated mechanosensation and mechanotransduction (*1*), the way cells sense compressive stress remains unclear. It has been demonstrated that MSICs, which are more easily activated in breast cancer cells compared to normal mammary cells (*19*), are activated in response to a range of forces, including those that stretch the cell membrane, leading to ion influx that stimulates intracellular signaling (*13*, *20*). Limited evidence suggests that some MSICs may also respond to mechanical loading and may be activated under conditions of heightened compressive force (*21*–*24*). To determine whether compression could be sensed by MSICs in breast cancer, we used the broad-specificity MSIC inhibitor mechanotoxin peptide 4 (GsMTx4) derived from the Chilean rose tarantula *Grammostola spatulata* (*25*, *26*). Enhancement of RhoA-ROCK signaling by compressive stress was suppressed by MSIC inhibition in both PyMT tumor fragments (Fig. 2A) and cells in collagen matrices (Fig. 2B). To identify the MSIC(s) responsible for sensing and transducing compressive mechanical signals in our system, we evaluated three candidate MSICs that are known to be activated in response to mechanical loading and lead to calcium signaling: Piezo1 and Piezo2 (*21*), which are inhibited by GsMTx4 (*25*, *26*), and transient receptor potential vanilloid 4 (Trpv4) (*24*, *27*). Independently, these candidates have been associated with Rho-ROCK signaling (*28*–*31*) often with conflicting observations reported, attributable to context-dependent functions. We have also previously demonstrated that while Piezo1 and Trpv4 both initiate mechanoelectrical transduction upon mechanical loading, these MSICs differ in their response times and mechanosensitivity (*24*). We therefore silenced all three MSICs using a small interfering RNA (siRNA) approach in primary PyMT mammary tumor cells and investigated the consequences for compressive stress–induced signaling (see schematic fig. S1D). Given concerns about the specificity of anti-PIEZO1 antibodies in the field (*32*), we first established the specificity of the anti-PIEZO1 antibody by immunohistochemical analysis (fig. S2A) of HEK-293T cells in which PIEZO1 had been depleted via a CRISPR-Cas9 approach (*33*) and immunofluorescence (fig. S2B) and Western (fig. S2C) analyses of primary mammary tumor cells in which Piezo1 had been depleted via siRNA. Depletion of all three candidate MSICs was verified by Western analysis for protein expression (fig. S2, C to E) and noted to be specific (fig. S2, C and F). We found that neither *Piezo2* nor *Trpv4* depletion affected compressive stress–induced RhoA-ROCK signaling (Fig. 2, C and D). A caveat to this conclusion is that while compressed cells in which *Piezo2* had been depleted exhibited higher pMlc2 and pMypt1 levels relative to their uncompressed controls, this difference was not statistically significant. We therefore cannot exclude the possibility that this difference occurred by chance. Pretreatment of PyMT cells with a specific inhibitor of Trpv4, HC067047, also did not have any effect on levels of RhoA-ROCK activity (fig. S2G). On the other hand, *Piezo1* knockdown strongly suppressed RhoA-ROCK signaling induced by compression (Fig. 2E). To support this observation, we took advantage of the HEK-293T model cell line, in which we had previously shown that compression could activate RHO-ROCK signaling (*10*). PIEZO1 knockout (KO) HEK-293T cells (*33*) (fig. S2A) did not exhibit compressive stress–induced elevation of RHOA-GTP or pMYPT1 (fig. S2H). Together, these data show that Piezo1 is required for compressive stress–induced RhoA-ROCK signaling.

To investigate whether compressive force–induced in vivo tumor growth is dependent on Piezo1, we subjected primary PyMT tumor cells in collagen matrices with and without *Piezo1* knockdown to compression at 20 kPa for 30 min, transplanted orthotopically into WT recipient mammary glands, and permitted them to grow for 4 weeks (see schematic fig. S2I). Measurement of gross tumor area at the end of this period showed that *Piezo1* depletion abolished tumor growth enhancement conferred by compression (Fig. 2F and fig. S2J). Together, these observations strongly suggest that in mammary tumors, Piezo1 is a mechanosensor for compressive stress, enhances RhoA-ROCK mechanotransduction signaling, and thereby promotes tumor growth.

Previous studies in load-bearing tissues have suggested that Piezo1 is up-regulated by compression (*34*–*36*), and we therefore investigated whether Piezo1 is regulated at the gene and/or protein levels over time in our system. PyMT cells in collagen matrices were subjected to 20-kPa compressive stress for 30 min and cultured for a further 2 hours or 24 hours, and *Piezo1* mRNA levels were analyzed by quantitative polymerase chain reaction (qPCR). No significant differences in *Piezo1* gene expression were observed between compressed and uncompressed samples either at the 2- or 24-hour time points (fig. S3A). To determine whether compression influenced Piezo1 protein levels, we used the ALginate Tissue ENcapsulation (ALTEN) biomimetic explant culture system, which we have previously characterized (*37*), to maintain tissue fragments in physiologically representative 3D culture conditions for several days following compression. Over 3 days of culture in ALTEN (see schematic fig. S3B), we did not see any changes in protein expression of Piezo1 (fig. S3C). This suggests that compressive stress–induced RhoA-ROCK mechanotransduction signaling in mammary cancer is likely not caused by enhanced Piezo1 levels.

We next examined PIEZO1 in human breast cancers. Using data from human patients with breast cancer from The Cancer Genome Atlas (TCGA) (*38*) compiled in the Oncomine online database (*39*), we found that *PIEZO1* was significantly higher in breast carcinoma and invasive breast carcinoma specimens compared to normal breast tissue (Fig. 2G). In invasive carcinoma specimens, median expression of *PIEZO1* mRNA was slightly lower than in noninvasive carcinoma samples, although this difference was not statistically significant (Fig. 2G). This observation was mirrored in mice; staged mammary tissue was extracted, encompassing normal gland (non-PyMT), hyperplasia (6-week-old PyMT^+^ mice; no palpable tumor), early tumor (8-week-old PyMT^+^ mice; palpable tumor contained within the mammary gland), and advanced tumor (10-week-old PyMT^+^ mice; large, externally visible multifocal tumors, throughout the mammary gland). Analysis of these tissues showed that levels of Piezo1 protein were higher in tumors compared to normal mammary epithelium yet slightly lower in more advanced tumors compared to early tumors while still being significantly higher than that in normal ducts (Fig. 2H). The fact that the PyMT mice effectively model the differences in Piezo1 expression levels across normal and cancer states observed in human specimens supports its use as an in vivo model to study the role of Piezo1 in mammary cancer while suggesting the interesting possibility that cells with high levels of PIEZO1 may have a selective advantage that permits them to predominate at early stages of tumorigenesis.

We then examined the correlation of PIEZO1 and ROCK proteins in tissue microarrays (TMAs) derived from human patients with breast cancer, as we have previously shown ROCK to be progressively overexpressed and activated in breast cancer and associated with invasiveness (*40*). There was a significant correlation between PIEZO1 and ROCK in in situ ductal and lobular carcinoma specimens (Fig. 2I), whereas in invasive ductal and lobular carcinoma specimens, this correlation was not significant (fig. S3D). When taken with the above findings (Fig. 2, G and H) and the fact that ROCK levels are progressively higher in invasive breast cancers relative to both normal breast tissue and hyperplastic breast (*40*), this suggests that high levels of PIEZO1 may be more strongly selected for at earlier stages of breast cancer, potentially when tumors are under high mechanical stress from compressive loads, and that signaling through ROCK is likely further enhanced by alternative mechanisms in invasive breast carcinoma. Accordingly, analysis of patient survival in the TCGA cohort using KM Plotter (*41*) suggests that high *PIEZO1* gene expression is associated with worse overall survival (fig. S3E).

To investigate whether human breast cancer cells also sense compressive stress via the same molecular mechanism, we applied compressive stress to the MCF7 and T47D patient-derived luminal breast cancer cell lines, both of which were observed to have PIEZO1 and basal ROCK activity levels comparable to those observed in PyMT cells (fig. S3F). Compression significantly increased signaling through the RHO-ROCK pathway, elevating levels of RHOA-GTP, pMLC2, and pMYPT1 (Fig. 2J and fig. S3G), suggesting that PIEZO1-mediated mechanosensation of compressive stress is also a feature of human breast cancer cells.

To ascertain whether our findings are unique to compressive force, we used the Flexcell FX-6000T Tension System to apply equiaxial static stretch at 10% strain to a layer of primary PyMT cells for 15 or 60 min. We found that the application of tension was also able to activate ROCK signaling, but that depleting *Piezo1* did not abolish whole-cell stretch–induced ROCK activation (fig. S3H), suggesting that Piezo1 likely does not mediate mechanosensation under these conditions. Therefore, Piezo1 appears to be a unique mechanosensor in mammary tumors, leading to activation of RhoA-ROCK signaling under specific conditions of compressive force. We then sought to bridge these findings by investigating how signals are transduced between Piezo1 and RhoA.

### Compressive stress–induced activation of mechanosensitive Piezo1 leads to downstream calcium-CaMKII-RhoA-ROCK signaling

**Fig. 3.**
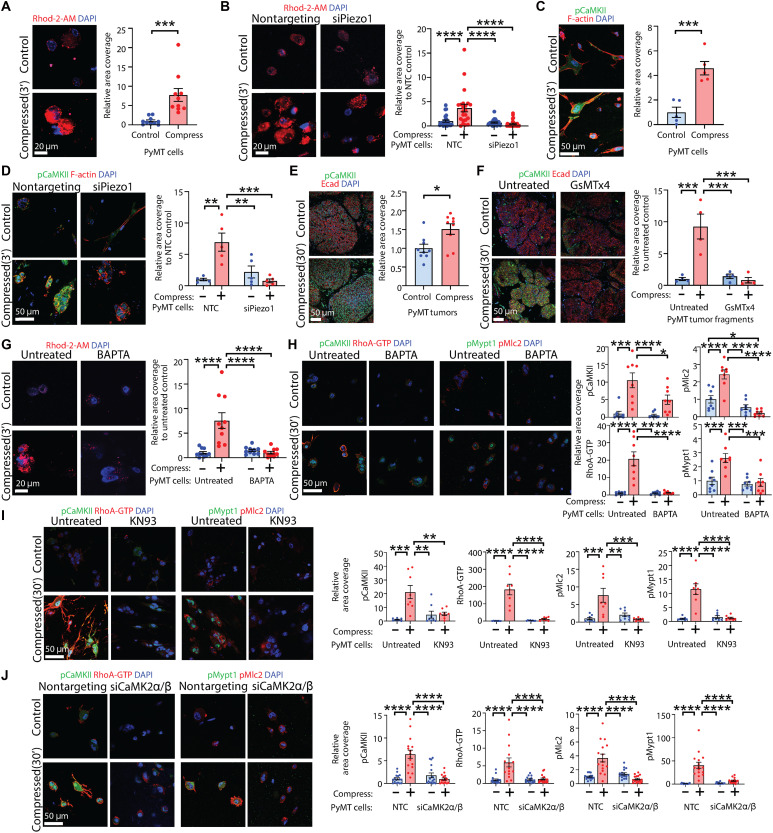
Compressive stress–induced activation of mechanosensitive Piezo1 leads to downstream calcium-CaMKII-RhoA-ROCK signaling. (**A**) Rhod-2-AM influx into tumor cells, compressed for 3 min. Scale bar, 20 μm. Chart: relative area coverage; mean ± SEM (*n* = 10 FOV per group); unpaired *t* test. (**B**) Rhod-2-AM influx into tumor cells ± *Piezo1* knockdown, compressed for 3 min. Scale bar, 20 μm. Chart: relative area coverage; mean ± SEM [*n* = 26 FOV nontargeting control (NTC) control, 22 NTC compressed, 25 siPiezo1 control, and 28 siPiezo1 compressed]; ANOVA. (**C**) Phospho-CaMKII (pCaMKII) in tumor cells, compressed for 3 min and colabeled for F-actin. Scale bar, 50 μm. Chart: relative area coverage; mean ± SEM (*n* = 5 FOV per group); unpaired *t* test. (**D**) pCaMKII in tumor cells ± *Piezo1* knockdown, compressed for 3 min and colabeled for F-actin. Scale bar, 50 μm. Chart: relative area coverage; mean ± SEM (*n* = 5 FOV per group); ANOVA. (**E**) pCaMKII in compressed tumor tissue, colabeled for E-cadherin. Scale bar, 50 μm. Chart: relative area coverage; mean ± SEM (*n* = 9 tumors per group); unpaired *t* test. (**F**) pCaMKII in tumor tissue pretreated with GsMTx4 and compressed, colabeled for E-cadherin. Scale bar, 50 μm. Chart: relative area coverage; mean ± SEM (*n* = 4 tumors per group); ANOVA. (**G**) Rhod-2-AM influx into tumor cells pretreated with 1,2-bis(2-aminophenoxy)ethane-*N*,*N*,*N*′,*N*′ tetraacetic acid (BAPTA), compressed for 3 min. Scale bar, 20 μm. Chart: relative area coverage; mean ± SEM (*n* = 10 FOV per group); ANOVA. (**H**) pCaMKII/RhoA-GTP (left) and pMlc2/pMypt1 (right) in tumor cells pretreated with BAPTA and compressed. Scale bar, 50 μm. Charts: relative area coverage; mean ± SEM (*n* = 8 FOV per group); ANOVA. (**I**) pCaMKII/RhoA-GTP (left) and pMlc2/pMypt1 (right) in tumor cells pretreated with KN93 and compressed. Scale bar, 50 μm. Charts: relative area coverage; mean ± SEM (*n* = 8 FOV per group); ANOVA. (**J**) pCaMKII/RhoA-GTP (left) and pMlc2/pMypt1 (right) in tumor cells ± siRNA targeting CaMKIIα and CaMKIIβ and compressed. Scale bar, 50 μm. Charts: relative area coverage; mean ± SEM (*n* = 15 FOV per group RhoA-GTP and 16 FOV per group pCaMKII/pMlc2/pMypt1); ANOVA.

Piezo channels are non-selective cation channels, and their opening leads to an influx of Ca^2+^ that can activate Rho-ROCK signaling (*42*). Because RhoA was activated within 5 min of compression (fig. S1E), we chose a 3-min time point to investigate Ca^2+^influx because we surmised that this would precede RhoA activation. Preincubation with the fluorescent Ca^2+^ indicator Rhod-2-AM revealed that Ca^2+^ influx is significantly elevated within 3 min of compression of PyMT tumor cells (Fig. 3A). Using three independent Ca^2+^ indicators (Rhod-2-AM, Cal520-AM, and Fluo8-AM), we found that compressive stress–induced influx of extracellular Ca^2+^ was blocked by *Piezo1* depletion (Fig. 3B and fig. S4A). These data suggest that the application of compressive force to these cells causes opening of the MSIC Piezo1, permitting Ca^2+^ influx.

Intracellular Ca^2+^ binds to calmodulin and can cause sustained activation of CaMKII via a conformational change in the binding subunit that permits (trans)autophosphorylation of two kinase subunits at Thr^286^, which allows the kinase to activate small GTPases including RhoA (*43*, *44*). We therefore evaluated Ca^2+^-mediated signaling activity by assessing the Thr^286^ phosphorylation status of CaMKII upon compression. In PyMT cells embedded in collagen matrices, CaMKII was activated within 3 min of compression (Fig. 3C), and this was abolished by *Piezo1* depletion (Fig. 3D). In tumor tissue subjected to compression for 30 min, activation of CaMKII was sustained (Fig. 3E), and pretreatment with GsMTx4 to block MSICs abolished this (Fig. 3F). Whereas knockdown of the other candidate MSICs *Piezo2* and *Trpv4* also reduced Ca^2+^ influx (fig. S4B), in contrast to *Piezo1* depletion, neither *Piezo2* nor *Trpv4* silencing affected compressive stress–induced activation of CaMKII (fig. S4C), consistent with the maintenance of RhoA-ROCK signaling (Fig. 2, C and D). As further support for the role of Piezo1 upstream of CaMKII-RhoA-ROCK, PyMT primary tumor cells were stimulated with the small molecule Yoda1, a specific agonist for Piezo1 (*45*, *46*). This resulted in activation of the entire signaling cascade, as shown by elevated phospho-CaMKII (pCaMKII), RhoA-GTP, pMlc2, and pMypt1 (fig. S4D).

To further strengthen the link between Ca^2+^ influx and RhoA-ROCK signaling downstream of compression, we chelated extracellular Ca^2+^ using non–cell-permeable 1,2-bis(2-aminophenoxy)ethane-*N*,*N*,*N*′,*N*′ tetraacetic acid (BAPTA) before compression, which blocked Ca^2+^ entry upon compression (Fig. 3G) and the complete compressive stress–induced CaMKII-RhoA-ROCK signaling cascade (Fig. 3H). We also assessed the impact of blocking CaMKII before compression. In a pharmacological approach, pretreatment of cells embedded in collagen with the CaMKII inhibitor KN93, which binds to and prevents the Ca^2+^-calmodulin complex from binding to CaMKII (*47*), inhibited compressive stress–induced CaMKII-RhoA-ROCK signaling (Fig. 3I). CaMKII has four isoforms, α, β, γ, and δ, all of which are expressed in human breast cancer (*48*). α and β isoforms are strongly associated with breast cancer aggressiveness and poor prognosis in patients (*48*, *49*). We therefore performed dual knockdown of both *Camk2a* and *Camk2b* in PyMT tumor cells (fig. S4E), embedded these cells in collagen matrices, and applied compressive force. Dual *Camk2a/Camk2b* depletion abolished compressive stress–induced RhoA-ROCK signaling (Fig. 3J).

Together with the above, these findings indicate that compressive force can be sensed by the MSIC Piezo1 in mammary cancer, which permits Ca^2+^ ion influx into the cell and calcium-mediated downstream signaling via the RhoA/ROCK pathway.

### Compressive stress–induced signaling influences malignant cellular properties of proliferation and epithelial-mesenchymal transition

**Fig. 4.**
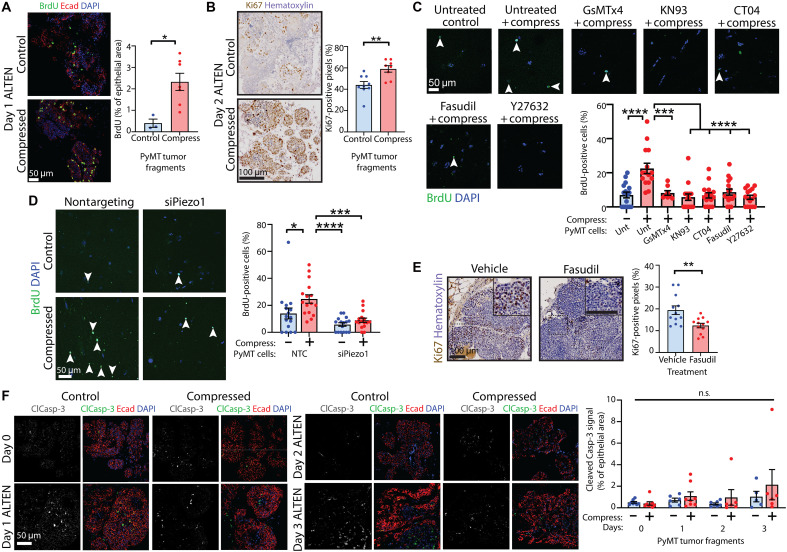
Compressive stress–induced signaling influences proliferation. (**A**) BrdU incorporation in compressed tumor tissue cultured in ALTEN, colabeled for E-cadherin. Scale bar, 50 μm. Chart: percentage of area coverage BrdU/epithelial area; mean ± SEM (*n* = 3 FOV control and 7 compressed); unpaired *t* test. (**B**) Ki67 in compressed tumor tissue cultured in ALTEN, counterstained with hematoxylin. Scale bar, 100 μm. Chart: percentage of positive pixels; mean ± SEM (*n* = 9 control and 8 compressed); unpaired *t* test. (**C**) BrdU incorporation in tumor cells treated with GsMTx4, KN93, CT04, Fasudil, or Y27632 and compressed. Scale bar, 50 μm. Chart: percentage of BrdU-positive cells; mean ± SEM (*n* = 16 FOV control/KN93/CT04/Fasudil/Y27632, 15 compressed, and 8 GsMTx4); ANOVA. (**D**) BrdU incorporation in tumor cells ± *Piezo1* knockdown and compression. Scale bar, 50 μm. Chart: percentage of BrdU-positive cells; mean ± SEM (*n* = 16 FOV per group); ANOVA. (**E**) Ki67 in tumors following engraftment of advanced PyMT tumor cells and subsequent in vivo Fasudil treatment, counterstained with hematoxylin. Scale bars, 100 μm. Insets show magnified views. Chart: percentage of positive pixels; mean ± SEM (*n* = 12 tumors per treatment); unpaired *t* test. (**F**) Cleaved Caspase-3 (ClCasp-3) in compressed tumor tissue cultured in ALTEN, colabeled for E-cadherin. Scale bar, 50 μm. Chart: percentage of relative area coverage of ClCasp-3/epithelial area; mean ± SEM (*n* = 8 tumor fragments day-0 control/day-0 compressed/day-1 compressed, 7 day-2 control, 6 day-1control/day-2 compressed/day-3 compressed, and 5 day-3 control); unpaired *t* tests (per day) and ANOVA (entire time course). *P < 0.05, **P < 0.01, ***P < 0.001, and ****P < 0.0001.

We next coupled PyMT primary mammary tumor cells embedded in collagen matrices with the ALTEN explant culture system, which reliably maintains epithelial and stromal architecture (*37*), to investigate whether compressive stress–induced mechanotransduction signaling influences cell and tissue function. When PyMT tumor tissue was subjected to compression and then cultured using ALTEN (see schematic fig. S3B), we found a significantly larger proliferative capacity as demonstrated by a larger percentage of cells incorporating 5-bromo-2′-deoxyuridine (BrdU) and more Ki67 labeling compared to uncompressed counterparts (Fig. 4, A and B), and this enhanced proliferative capacity was maintained for an extended period (at least 2 days following acute compression). In PyMT tumor cells (see schematic fig. S5A), enhanced proliferation induced by compression was abolished by inhibiting MSICs, CaMKII, Rho, or ROCK (Fig. 4C), silencing *Piezo1* (Fig. 4D) or dual silencing of *Camk2a*/*Camk2b* (fig. S5B). These observations complement our findings in the in vivo transplant model (Fig. 1, A to C), where compressive force enhanced Ki67 positivity and caused tumors to grow larger than uncompressed controls. Together, these data show that compression-mediated activation of Piezo leading to calcium influx and Rho-ROCK signaling mediates compression-induced proliferation and tumor growth in this model. Consistent with these data, tumors from mice that had been orthotopically engrafted with cancer cells from advanced PyMT tumors that we have previously shown to exhibit activation of endogenous ROCK (*40*) had low levels of proliferation when injected with Fasudil, a pharmacological inhibitor of ROCK (Fig. 4E), showing that ROCK activity enhances proliferation in PyMT^+^ mammary tumors. In contrast to previous studies conducted in skin or load-bearing tissues (*34*, *36*, *50*), we did not observe more apoptosis in tumors grown from transplantation of compressed tumor tissue (fig. S1B) or explants subjected to compression and cultured using the ALTEN system for up to 3 days (Fig. 4F). These data suggest that enhancement of cell proliferation caused by compressive stress is mediated by Piezo1 and calcium signaling via RhoA-ROCK and may explain the enhanced growth of tumors following compression.

**Fig. 5.**
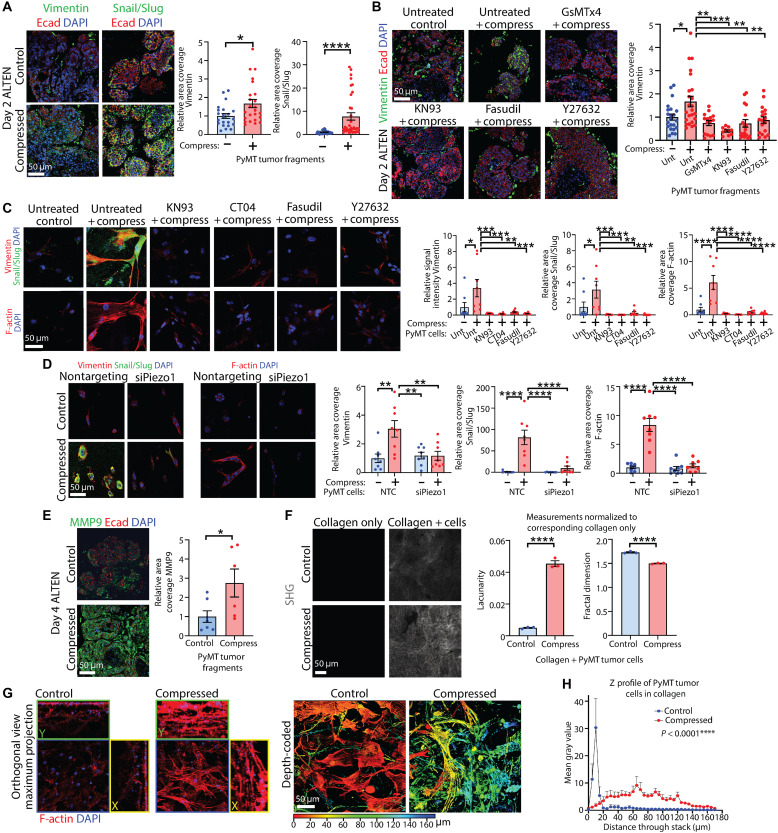
Compressive stress–induced signaling influences EMT. (**A**) Vimentin and Snail/Slug in tumor tissue compressed and cultured in ALTEN, colabeled for E-cadherin. Scale bar, 50 μm. Charts: relative area coverage; mean ± SEM (Vimentin: *n* = 22 FOV control, 24 compressed. Snail/Slug: *n* = 32 FOV control, and 34 compressed); unpaired *t* tests. (**B**) Vimentin in tumor tissue untreated (Unt) or pretreated with GsMTx4, KN93, Fasudil, or Y27632, compressed and cultured in ALTEN and colabeled for E-cadherin. Scale bar, 50 μm. Chart: relative area coverage; mean ± SEM (*n* = 22 FOV control, 24 compressed, 15 GsMTx4, 10 KN93, 17 Fasudil, and 18 Y27632); ANOVA. (**C**) Vimentin and Snail/Slug (top), and phalloidin labeling of F-actin (bottom) in tumor cells pretreated with KN93, CT04, Fasudil, or Y27632, compressed and cultured overnight. Scale bar, 50 μm. Charts: relative intensity (Vimentin) and area coverage (Snail/Slug and F-actin); mean ± SEM (*n* = 8 FOV per group); ANOVA. (**D**) Vimentin and Snail/Slug (left), and phalloidin labeling of F-actin (right) in tumor cells ± *Piezo1* knockdown, compressed and cultured. Scale bar, 50 μm. Charts: relative area coverage; mean ± SEM (*n* = 8 FOV per group); ANOVA. (**E**) Matrix metalloproteinase 9 (MMP9) in compressed tumor tissue cultured in ALTEN, colabeled for E-cadherin. Scale bar, 50 μm. Chart: relative area coverage; mean ± SEM (*n* = 7 FOV control and 6 compressed); unpaired *t* test. (**F**) Second harmonic generation (SHG) from collagen matrices ± PyMT tumor cells and compression. Tumor cells were allowed to remodel collagen for 4 days before imaging. Scale bar, 50 μm. Charts: lacunarity and fractal dimension of collagen + cells, calculated using The Workflow Of Matrix BioLogy Informatics (TWOMBLI) and normalized to corresponding collagen only (−cells) samples; mean ± SEM (*n* = 3 matrices + cells per group and average 3 FOV per matrix); unpaired *t* tests. (**G**) Confocal z-stack images of cells in collagen, compressed and cultured for 4 days. Cells labeled with phalloidin (F-actin) and DAPI. Images show orthogonal views of maximum projections (left) and depth-coded images of the z-stacks (right). (**H**) Graph: Z-profile analysis of stacks in (G), showing mean gray value per slice over a 170-μm range. Mean + SEM (*n* = 6 FOV per group); two-way ANOVA. *P < 0.05, **P < 0.01, ***P < 0.001, and ****P < 0.0001.

We next examined the effects of compression on epithelial-mesenchymal transition (EMT), a critical driver of cancer invasion and metastasis. The mesenchymal marker Vimentin and EMT-regulating transcription factors Snail (Snai1) and Slug (Snai2), assessed using an antibody recognizing both transcription factors, were all significantly higher in compressed PyMT tumor tissue in the epithelial compartment after 2 days in ALTEN ex vivo culture (Fig. 5A), and this was not observed in tumor fragments that had been pretreated with inhibitors against MSICs, CaMKII, or ROCK (Fig. 5B and fig. S5C). Vimentin and Snail/Slug were also elevated in PyMT cells subjected to compression within collagen matrices, and this elevation was blocked by pretreatment with inhibitors of CaMKII, Rho, or ROCK (Fig. 5C) or when *Piezo1* or *Camk2a*/*Camk2b* were depleted (Fig. 5, D and E, and fig. S5D). In addition, we observed that compression increased levels of filamentous (F)–actin, an indicator of acquired mesenchymal phenotype and enhanced migratory capacity due to cytoskeletal rearrangement (*51*). As with Vimentin and Snail/Slug, elevation of F-actin by compression was blocked by pharmacological inhibition at any step of the pathway or by *Piezo1* knockdown (Fig. 5, C and D). Together, these findings strongly suggest that compressive force induces malignant cellular properties of proliferation and EMT in a manner that requires Piezo1, calcium, and RhoA-ROCK signaling.

To functionally demonstrate that compression induced EMT in primary mammary cancer cells, we examined the effects of compression on ability of cancer cells to invade and modify their matrix environment. Compressed tumor tissue cultured in ALTEN for 4 days exhibited significantly higher levels of the matrix remodeling enzyme matrix metalloproteinase 9 (MMP9) compared to controls (Fig. 5E), and isolated cancer cells embedded in collagen and subjected to compression had elevated levels of ECM genes (fig. S5E), typical of cells that have undergone an EMT (*52*). Using second harmonic generation (SHG) imaging, we then assessed remodeling of collagen by PyMT tumor cells that had been subjected to compression and then maintained in culture for 4 days (Fig. 5F). The Workflow Of Matrix BioLogy Informatics (TWOMBLI) (*53*) analysis of collagen SHG showed that matrices in which mammary cancer cells subjected to compression had been maintained exhibited higher collagen lacunarity, signifying more gaps and structural heterogeneity and indicating a pruned and reorganized structure, and lower fractal dimension, indicating low pattern complexity (Fig. 5F, right). Together, these observations suggest that primary mammary cancer cells subjected to compression were consequently more capable of remodeling a collagen matrix. To investigate compression-induced changes in invasive capacity, we compressed primary mammary cancer cells within collagen matrices and maintained them in culture for 4 days. We then fluorescently labeled their F-actin and conducted 3D confocal fluorescence imaging (fig. S5F). We observed elevated F-actin in compressed cells, consistent with our earlier findings (Fig. 5, C and D). Orthogonal views along the imaging *z* axis suggested that compressed cells were able to invade more deeply into the collagen matrix (Fig. 5G, left). We therefore processed the 3D image stacks to produce depth map coding (Fig. 5G, right). These analyses revealed that compressed cells had invaded more extensively into the matrix than cells that had not been compressed, as indicated by a large number of cells located at a greater depth compared to control cells, which were, in contrast, localized more superficially within the matrices (Fig. 5H and fig. S5F). These data suggest that compressed cancer cells were significantly more invasive than cells that had not been compressed.

Because PIEZO1 levels peak early in mouse and human tumors (Figs 2, G and H) and then dwindle at more advanced stages and PIEZO1 and ROCK are correlated significantly in in situ carcinomas but not in invasive carcinomas (Fig. 2I and fig. S3D), we wondered whether cells from early- (7 to 8 weeks old) and late- (10 weeks) stage PyMT tumors would exhibit divergent functional consequences to compression. We found that compression of cancer cells from late-stage tumors enhances neither proliferation (fig. S5G) nor EMT (fig. S5H) to the extent it does in cancer cells derived from early-stage tumors. In addition, unlike in cancer cells derived from early tumors, there was no statistically significant difference in proliferation or EMT marker levels when *Piezo1* was silenced. These observations strongly suggest that Piezo1 has a key role in mechanosensing compressive stress at early stages of mammary tumorigenesis but is functionally less important at later stages.

### Compressive stress results in posttranslational histone modifications

**Fig. 6.**
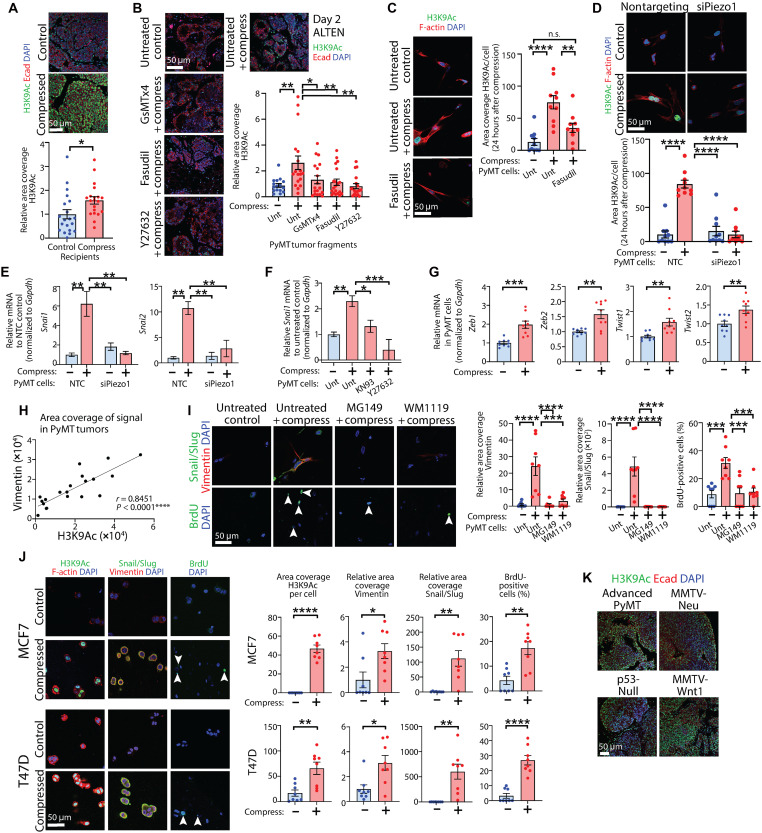
Compressive stress results in posttranslational histone modifications. (**A**) H3K9Ac in tumors formed from transplantation of compressed tumor tissue, colabeled for E-cadherin. Scale bar, 50 μm. Chart: relative area coverage; mean ± SEM (*n* = 20 tumors per group); unpaired *t* test. (**B**) H3K9Ac in tumor tissue pretreated with GsMTx4, Fasudil, or Y27632, compressed, cultured in ALTEN, and colabeled for E-cadherin. Scale bar, 50 μm. Chart: relative area coverage; mean ± SEM (*n* = 16 FOV control, 18 compressed/GsMTx4/Y27632, 20 Fasudil); ANOVA. (**C**) H3K9Ac in tumor cells treated with Fasudil, compressed, cultured overnight, and colabeled for F-actin. Scale bar, 50 μm. Chart: area coverage per cell; mean ± SEM (*n* = 10 FOV per group); ANOVA. (**D**) H3K9Ac in tumor cells ± *Piezo1* knockdown, compressed, cultured overnight, and colabeled for F-actin. Scale bar, 50 μm. Chart: area coverage per cell; mean ± SEM (*n* = 10 FOV per group); ANOVA. (**E**) *Snai1* and *Snai2* expression in compressed tumor cells ± *Piezo1* knockdown. Charts: relative mRNA; mean ± SEM (Snail: *n* = 3 per group; Slug: *n* = 3 NTC/siRNA control and 2 NTC/siRNA compressed); ANOVA. (**F**) *Snai1* expression in tumor cells treated with KN93 or Y27632 and compressed. Chart: relative mRNA; mean ± SEM (*n* = 6 control/compressed and 3 KN93/Y27632); ANOVA. (**G**) *Zeb1*, *Zeb2*, *Twist1*, and *Twist2* expression in compressed tumor cells. Charts: relative mRNA; mean ± SEM (*n* = 9 per group); unpaired *t* tests. (**H**) Graph: H3K9Ac versus Vimentin quantitative immunofluorescence in advanced tumors (*n* = 20), with Spearman correlation coefficient, two-sided test. (**I**) Vimentin, Snail/Slug, and BrdU, in tumor cells treated with inhibitors of Tip60 (MG149) or KAT6A (WM1119), compressed and cultured overnight. Scale bar, 50 μm. Charts: relative area coverage of Vimentin/Snail/Slug and percentage of BrdU-positive cells; mean ± SEM (*n* = 8 FOV per group); ANOVA. (**J**) H3K9Ac (colabeled for F-actin), Vimentin and Snail/Slug, and BrdU in MCF7 and T47D human breast cancer cells, compressed and cultured overnight. Scale bar, 50 μm. Charts: area coverage per cell H3K9Ac, relative area coverage of Vimentin/Snail/Slug, and percentage of BrdU-positive cells; mean ± SEM (*n* = 8 FOV per group); unpaired *t* tests. (**K**) H3K9Ac in advanced tumors from breast cancer mouse models, MMTV-PyMT, MMTV-Neu, p53-Null, and MMTV-Wnt1, colabeled for E-cadherin. Scale bar, 50 μm.

As we had observed long-lasting (up to 4 weeks, in the case of in vivo transplants) and persistent biological effects in cells and tissues following acute compression, including increased proliferation (Figs. 1C and 4, A to D), promotion of EMT (Fig. 5), and enhanced tumor growth in vivo (Figs. 1, A and B, and 2F), we wondered whether the cue of acute compression may be priming mammary cancer cells to develop mechanical memory (*54*). Recent developments have suggested that chromatin structure underpins mechanoreciprocity and that cellular adaptations to mechanical environments are regulated via epigenetic modifications (*12*, *55*). To address whether compression results in chromatin changes, we assessed cells in collagen, ALTEN explants, and in vivo tumors formed from transplantation for posttranslational histone 3 (H3) epigenetic modifications on lysine residues (*56*) following application of compression. Assessment of global acetylation marks associated with open euchromatin (*57*) using an antibody that detects acetylation on H3 lysines (H3K) at positions 4, 9, 14, 18, 23, and 27 revealed that levels of H3K acetylation (H3KAc) were higher upon compression in all three models [in vivo (schematic fig. S1A), explant (schematic fig. S3B), and cells, in which cells were examined 24 hours following compression (schematic fig. S5A)] (fig. S6, A to C). This higher H3KAc was not observed following pretreatment with inhibitors of MSICs or ROCK in explants and cells (fig. S6, B and C) or if *Piezo1* was depleted in cells (fig. S6D). This suggests that compressive stress–induced mechanotransduction may regulate a wide range of posttranslational histone modifications associated with transcriptionally active chromatin. We then looked more specifically at the active histone mark H3K 9 acetylation (H3K9Ac) that has been extensively implicated in promoter activation and gene transcription and associated with shorter recurrence-free survival in patients with breast cancer (*58*). We found that, as with the broader analysis above, levels of H3K9Ac were significantly higher upon compression in all three models (Fig. 6, A to C) but not when samples had been pretreated with inhibitors of MSICs or ROCK (Fig. 6, B and C) or *Piezo1* knockdown (Fig. 6D and fig. S6E). These acetylation marks were persistent, lasting for at least 2 days in explants (Fig. 6B) and cells (fig. S6E) and for at least 4 weeks in vivo (Fig. 6A).

To determine how rapidly changes in H3K9Ac occur, we next examined PyMT cells at 2 and 4 hours postcompression. We found that acetylation on H3K9 was significantly enhanced compared to uncompressed control cells even at the early 2-hour time point and enhanced further at 4 hours (fig. S6F). Accordingly, we then sought to evaluate transcriptional changes 2 hours following compression, as the increased acetylation at this early time point suggested that chromatin had already been made accessible by compression. In line with our findings in this study (Fig. 5), it is documented that histones can be acetylated to facilitate EMT (*59*). The most well documented of these are Snail and Slug, which are subject to promoter H3K9Ac (*60*–*62*). We observed that gene expression of *Snai1* and *Snai2* was enhanced by application of compression (Fig. 6, E and F), and this increase was prevented when *Piezo1* was knocked down (Fig. 6E) or CaMKII or ROCK inhibited (Fig. 6F). Promoters of other EMT regulatory genes are also known to be occupied by acetylated H3, including on K9 (*59*, *63*), and we found that *Zeb1*, *Zeb2*, *Twist1*, and *Twist2* were all transcriptionally induced by compression (Fig. 6G). Further strengthening the relationship between H3K9Ac and changes in tumor phenotype, examination of advanced PyMT tumors, which we have previously demonstrated to exhibit high levels of endogenous ROCK activity (*40*), showed a strong positive correlation between H3K9Ac and mesenchymal marker Vimentin (Fig. 6H). Activation of Rho (using Rho activator CN03 ex vivo) or ROCK [using an in vivo model whereby a ROCK:ER construct under control of a K14 promoter sequence is activated upon administration of tamoxifen (*40*)] phenocopied the application of compressive stress, enhancing H3K9Ac and EMT in tumor cells and tissues, respectively (fig. S6, G and H).

Given the role of histone acetyltransferases (HATs) in histone lysine acetylation, we inhibited two separate HATs, Tip60 (*64*) (using inhibitor MG149) and Kat6a (*65*) (using inhibitor WM1119), which acetylate K9. Both inhibitors blocked compressive stress–induced H3K9Ac in tumor tissue (fig. S6I). The downstream induction of EMT and proliferation were abolished when either HAT was pharmacologically inhibited (Fig. 6I). Conversely, inhibiting histone deacetylase 6 in PyMT tumor cells using Ricolinostat (*66*) elevated H3K9Ac independently of compression and increased Vimentin expression and proliferation (fig. S6J). We also observed lower H3K27 trimethylation, which is associated with closed (hetero) chromatin, in all three models (in vivo, explant, and cells), suggesting the potential for gene derepression by compressive stress (fig. S6K).

Last, we subjected human breast cancer cell lines MCF7 and T47D to compressive stress and found that in concert with our findings in the PyMT mouse model, compressive stress significantly enhanced H3K9Ac, induced EMT, and promoted proliferation (Fig. 6J). Furthermore, advanced tumors from three other genetic mouse models of mammary cancer (MMTV-Neu, p53-Null, and MMTV-Wnt1), which all show activation of the ROCK pathway (*40*), exhibited comparable levels of H3K9Ac to those seen in advanced tumors from the PyMT model (Fig. 6K), suggesting that our observations are not restricted to the PyMT model but are relevant across mammary cancers driven by different genetic lesions and in both mice and humans. Our observations therefore permit us to propose that compressive stress activates Piezo1 and ROCK to induce rapid and long-lasting epigenetic alterations, leading to an EMT and an ensuing mechanical memory to promote cancer progression.

## DISCUSSION

Mechanical force, both internally generated and externally applied, strongly influences tumor biology—most importantly, malignant potential (*6*). The ability to recapitulate force application ex vivo to study downstream effects is essential for a precise understanding of the mechanisms by which it directs tumor biology (*12*). Here, we applied physiologically relevant levels of compressive stress (*67*–*69*) to mammary cancer cells suspended in a physiologically relevant biomaterial, or to intact mammary tumors and tumor fragments within their in situ architectural contexts, to study the role of mechanical force in cancer progression. We showed here that compressive stress activates the Piezo1 MSIC, which stimulates Rho-ROCK signaling via extracellular Ca^2+^ influx, mediated via CaMKII. We found that this is not a general function of MSICs, as other known MSICs, Piezo2 and Trpv4, did not transduce compressive force to Rho-ROCK signaling. The compressive stress–induced Piezo1-Ca^2+^-CaMKII-Rho-ROCK pathway has substantial tumor-promoting capacity, including causing EMT, inducing a functional mesenchymal phenotype, and enhancing proliferation, ultimately driving tumor growth in vivo.

We found that the role of Piezo1 in activating ROCK signaling in primary mammary cancer cells may be specific to compressive stress, as its knockdown did not affect ROCK activity induced by the application of tensile stress, highlighting the need to carefully distinguish between the effects of compressive stress and tensile stress applied to this system. Given the variety of different mechanical inputs that have been demonstrated to be capable of activating Piezo1 (*70*–*73*) and the similarly diverse functional consequences (*74*, *75*), we are mindful that the context within which PIEZO1 is activated can determine signaling and functional outcomes and that more research is required before general principles of PIEZO1 mechanosensation with in vivo relevance can be established. Our observation that Piezo1 likely does not initiate Rho-ROCK–mediated mechanotransduction under conditions of whole-cell stretch contrasts with findings in endothelial cells (*76*, *77*) and chondrocytes (*78*), suggesting a tissue and/or cancer context-dependent mechanism, where differences may arise from the expression of different combinations of ion channels that have distinct activation profiles. The MSIC TRPV4 was activated in glaucoma trabecular meshwork cells subjected to tensile stretch using the same system, leading to Rho-ROCK–dependent cytoskeletal and focal adhesion remodeling (*79*); this has yet to be examined in mammary cancer.

In this study, we uncovered a fundamental mechanism by which compressive stress is transduced into biological outcomes in mammary cancer using a suite of pharmacological inhibitors and gene silencing, coupled with 3D models encompassing ex vivo cell culture, explant, and in vivo tumor growth. Our findings provide a mechanistic basis in a physiologically relevant 3D context, for previous observations made in 2D cell line systems linking uniaxial compressive stress to calcium influx through PIEZO1 (*80*) and compression using a weighted piston to enhanced cell migration (*81*), by showing that compressive stress–induced mechanotransduction leads to rapid histone modifications and tumor promotion that is persistent. This collectively suggests that the application of compressive stress engenders epigenetic mechanical memory.

Previous studies of mechanical memory have primarily focused on functional cellular responses to reseeding between soft and stiff substrates, which has been shown to regulate EMT (*82*), cell migration (*83*, *84*), and collagen remodeling and invasion (*85*). Other modes of mechanical stimuli induce condensation into transcriptionally silent heterochromatin (*86*–*88*), potentially a mechanoprotective response; however, our unconfined 3D system promotes increased euchromatin. As epigenetic modifications are key regulators of mammary lineage specification (*89*), it is plausible that these may also define breast cancer subtypes, influenced by compression at the early stages of tumor development when PIEZO1 levels are at their peak. Breast cancer tissue transitioning from a solid “jammed” to liquid-like “unjammed” state during invasion exhibits substantial changes in heterochromatin distribution (*90*), suggesting that cancer cells moving from the early stages of compressive stress–inducing tumor growth within the duct to invasiveness may undergo subsequent tumor-promoting modifications to their epigenome that allow them to withstand new stresses and adapt to dynamic mechanical environments dependent on space and time.

We have previously demonstrated that activation of ROCK in mammary cancer cells results in paracrine production of factors that reprogram stromal fibroblasts to a protumor form that remodels the ECM to become tumor permissive via selective activation of the PERK arm of the unfolded protein response (*40*). Consistent with this, cartilage cells under compressive load elevate Rho-ROCK–regulated production of MMPs and collagens (*15*, *91*), PIEZO1 activation leads to ECM production and remodeling in neural progenitor cells and in glioblastoma (*92*, *93*), and so mechanical perturbations may foster further production of ECM and ECM remodeling proteins, as we have observed in this study. Compression can skew macrophage polarization to an M2 phenotype via hyperacetylation of H3 (*94*), suggesting that the effects of compressive forces on stromal and immune cells may also contribute to cancer progression. Assessing the impact of compression on individual stromal and immune cell compartments and secondary impacts on ECM composition and architecture from these compartments, as well as cancer cells that have undergone EMT, requires further investigation.

It has been suggested that compression can decrease cellular migration, as well as suppress malignancy in breast cancer cells and reduce in vivo mammary tumor growth (*80*, *95*, *96*), and that, in turn, in vivo biomechanical intervention could harbor translational potential (*95*). Our study, using early-stage tumor cells that are likely noninvasive but potentially poised for EMT—a notion that is supported by our finding that compression had a greater impact on proliferation and EMT in cancer cells from early-stage tumors than in those from late-stage tumors—therefore serves as a warning that enhancement of exogenous compressive force may hasten malignancy in early cancers. Consequently, given the complications attendant on directly targeting ROCK activity in cancer therapy (*97*), we propose that the PIEZO1-CAMKII-RHO-ROCK axis should be evaluated as a mechanotherapy target, with potential utility at very early stages or in those with relevant susceptibilities.

## MATERIALS AND METHODS

### Experimental design

This study was designed to investigate the role of compressive stress on cancer biology. This was modeled using equipment that permitted the precise application of compressive force for a specified duration to primary cancer cells and tissues. The consequences of compressive force for cancer biology were investigated by culturing of cells and tissues subjected to compression ex vivo for their assessment by histological, immunohistochemical, biochemical, and molecular means; and by transplantation into immune competent mice for the assessment of growth and progression, as well as further assessment by histological, immunohistochemical, biochemical, and molecular means, for the establishment of causal and targetable relationships. Human patient samples were investigated for correlation with these observations and for the establishment of clinical relevance of our conclusions. Detailed procedures appear below.

### Human patient samples

Human breast TMA #1006 was obtained from Protein Biotechnologies Incorporated. The TCGA Breast gene expression dataset (*38*) was used in this study. Interrogation of human patient datasets was undertaken using the Oncomine (*39*) and KM Plotter (*41*) online tools. Work on human samples was conducted under appropriate licenses and with the oversight of the human research ethics committee of the Central Adelaide Local Health Network (CALHN; approval number HREC/16/RAH/163) and the University of South Australia (200774) Human Research Ethics Committee.

### Mouse models and in vivo procedures

The MMTV-PyMT line was maintained on a pure FVB/n strain background. All animal experiments were approved by the CALHN/SA Pathology (approval 25/16) and the University of South Australia (U09-21 and U10-25) Animal Ethics Committees, and this research is fully compliant with all relevant ethical regulations regarding animal research in South Australia. Female mice were used in this study. Unless specified, tumors used were early stage (still contained within the mammary gland, not invasive or necrotic), derived from mice at 7 to 8 weeks of age. Where late-stage tumor cells are indicated, these were derived from mice at 10 weeks of age.

K14-ROCK:mER;MMTV-PyMT (ROCK-PyMT) and K14-KD:mER;MMTV-PyMT (KD-PyMT) mice were bred and used as previously described (*40*). Mice were administered tamoxifen (60 mg/kg of bodyweight in corn oil; Sigma-Aldrich) twice, intraperitoneally at the ages of 8 and 9 weeks old, respectively, and tumors were harvested at 10 weeks old for analysis. Formalin-fixed and paraffin-embedded (FFPE) sections from other mouse mammary cancer models (MMTV-Neu, p53-Null, and MMTV-Wnt1) were a kind gift from J. Visvader (Walter and Eliza Hall Institute).

For transplantation experiments, PyMT tumor tissue (a) or PyMT tumor cells embedded in collagen (b) was subjected to compressive stress before transplanting 1-mm^3^ tissue fragments (a) or 500,000 cells within collagen (b) into the fourth inguinal mammary fat pads of anesthetized WT FVB/n recipient mice aged 6 to 9 weeks. Wounds were closed using surgical staples. After a maximum of 4 weeks, mice were humanely euthanized, and mammary glands were dissected out for assessment of cancerous lesions.

For engraftment experiments where mice were treated with Fasudil, 50,000 donor PyMT cells from advanced mammary tumors (mice at 10 weeks old) were resuspended in 20 μl of cold 20% Matrigel (Corning) prepared in Dulbecco’s modified Eagle’s medium (DMEM) and engrafted into the fourth inguinal mammary fat pads of anesthetized WT FVB/n recipient mice aged 6 to 8 weeks. Tumors were grown for 4 weeks, mice were humanely euthanized, and tumors were dissected out and fixed in formalin. In vivo Fasudil treatments were undertaken by injecting mice intraperitoneally with Fasudil monohydrochloride (25 mg/kg of bodyweight; Selleck Chemicals) or phosphate-buffered saline (PBS) vehicle once a week, 1 week following mammary tumor cell engraftment until the end point of the experiment.

### Application of compressive force and whole-cell stretch

The Flexcell FX-5000 Compression System was used to apply compressive force to whole tissues or cells embedded in collagen. Using BioPress Compression 6-well plates (Flexcell Int. Corp.), the application of compression was carried out as described previously (*10*). Compressive loads were applied using a static waveform at 20 kPa, and unless specified, compression was carried out for 30 min. Immortalized cells were subjected to the same compression regimen as for primary cells. All control samples were maintained throughout in an unloaded state.

The Flexcell FX-6000T Tension System was used to apply equiaxial tension to whole cells cultured on BioFlex Culture 6-well collagen I-coated plates (Flexcell Int. Corp.). Cells were seeded at 4 × 10^4^ per well 1 day before the application of tension and then starved for 4 hours before static strain of 10% at 1.0 Hz was applied for 15 or 60 min. Control cells were also seeded in BioFlex plates but maintained unstretched.

### Primary tumor cell derivation and culture of primary and immortalized cells

Primary mammary tumor cells were isolated as previously described (*98*). Freshly isolated total cell preparations were cultured for 2 days under nonadherent conditions [1:1 volumes of DMEM and Ham’s F12 nutrient mix (Thermo Fisher Scientific), supplemented with epidermal growth factor (10 ng/ml), basic fibroblast growth factor (20 ng/ml), and 0.5× B27 (Thermo Fisher Scientific)] to obtain a pure tumor cell culture. Cells were then cultured adherently in DMEM (Sigma-Aldrich) supplemented with 10% fetal bovine serum (FBS), with antibiotic-antimycotic (Thermo Fisher Scientific), at 37°C in an atmosphere of 5% CO_2_.

HEK-293T WT and CRISPR-Cas9 PIEZO1 KO cells [generated previously (*33*)], MCF7 cells, and T47D cells were cultured adherently as for primary PyMT mammary tumor cells. All immortalized cell lines were tested regularly and confirmed to be mycoplasma free.

Once embedded in collagen (see below), cells were immediately incubated in starvation medium (DMEM; no FBS) for 3 hours before compression. Culture following compression was performed in starvation medium (2- to 4-hour culture following compression) or DMEM/5% FBS (overnight). For invasion experiments only, culture was performed in starvation medium for 4 days.

### siRNA knockdown

Primary mammary tumor cells in culture were transfected using Lipofectamine RNAiMAX (Thermo Fisher Scientific) with nontargeting control (NTC) siRNA (Thermo Fisher Scientific) or siRNA targeting *Piezo1*, *Piezo2*, *Trpv4* (FlexiTube Gene Solution, QIAGEN), *Camk2a*, or *Camk2b* (siGENOME SMARTpool, Dharmacon). siRNAs were used at a final concentration of 10 nM (5 nM each Camk2α and Camk2β in dual knockdown). Tumor cells for in vivo studies underwent two rounds of transfection 24 hours apart (reverse transfection and then forward), and cells were collected, embedded in collagen, starved, compressed, and transplanted orthotopically 72 hours after the first transfection. For ex vivo studies, tumor cells underwent one round of transfection and were used at 72 hours posttransfection.

### Embedding cells in rat tail collagen

Single-cell preparations were embedded in a cocktail of 55% rat tail collagen (0.4 mg/ml in 17.5 mM acetic acid), 20% cold 1× PBS, 1× minimal essential medium (Thermo Fisher Scientific), and 10% FBS. The pH was adjusted using 0.22 M NaOH (~5% of cocktail) to approximately 7 as indicated by phenol red color change. Collagen matrices in 24-, 12-, or 6-well plates as appropriate were allowed to set for 10 min at 37°C before overlaying with starvation medium and gently pulling the collagen matrix away from the well walls using the pipette tip. Embedded cells were subjected to application of compression on the same day, following 3 hours of starvation.

### Collagen remodeling assessment by SHG and TWOMBLI

To assess remodeling of collagen by PyMT cells, cells were embedded in thick rat tail collagen matrices in 12-well plates. Cells in collagen were starved and compressed as above and then placed in serum-free DMEM for 4 days at 37°C. Matrices were then fixed in formalin for 20 min. SHG from collagen matrices was imaged using a ×20, 1.0 numerical aperture water immersion objective on an inverted two-photon laser scanning microscope system (Leica). The excitation source was a Ti:Sapphire femtosecond laser cavity (Chameleon Ultra, Coherent Scientific) coupled to an SP8 scan module. An excitation wavelength of 890 nm was used to collect SHG signal (435 ± 20 nm) as previously described (*8*, *99*) from fibrillar collagen. Structural assessments of collagen SHG images were conducted using TWOMBLI plugin (*53*) for the Fiji implementation of ImageJ (*100*).

### Inhibitors, agonists, labels, and indicators

To assess calcium influx in cells embedded in collagen, culture medium was removed and replaced with starvation medium containing Rhod-2-AM (4 μM), Cal520-AM (1 μM), or Fluo8-AM (4 μM) fluorescent Ca^2+^ indicators (all from Abcam) for 1 hour in the dark before compression.

The following inhibitors for cells and tissue fragments were used in this study at the final concentrations indicated: GsMTx4 (Abcam; 2.5 μM), HC067047 (Abcam; 0.05, 0.5, and 1 μM), BAPTA (Selleck Chemicals; 5 μM), KN93 (Selleck Chemicals; 20 μM), Exoenzyme C3 Transferase (CT04, Cytoskeleton Inc.; 1 μg/ml), Fasudil (Selleck Chemicals; 50 μM), Y27632 (Tocris Bioscience; 10 μM), MG149 (Selleck Chemicals; 74 μM), WM1119 (Selleck Chemicals; 0.25 μM) and Ricolinostat (Selleck Chemicals; 10 μM). In all cases, inhibitors were added in starvation medium for 3 hours before compression, fixation, or further culture.

The Piezo1 agonist Yoda1 (Sigma-Aldrich) was used at 20 μM, and cells were stimulated for 5 min following a 3-hour starvation. The Rho Activator CN03 (Cytoskeleton Inc.) was used at 1 μg/ml, and cells were treated for 3 hours in starvation medium. Cells for signaling analysis were fixed or lysed immediately following treatment, and cells for outcome analyses were fixed or lysed following overnight culture in DMEM/5% FBS.

For assessment of proliferation by BrdU incorporation, cells embedded in collagen or tissue encapsulated in alginate were pulsed with Cell Proliferation Labelling Reagent (Merck), consisting of an aqueous solution of 5-bromo-2-deoxyuridine and 5-fluoro-2′-deoxyuridine, at a dilution of 1:500 for 1 hour before fixation.

### ALginate Tissue ENcapsulation

Alginate encapsulation was performed as previously described (*37*). Briefly, PyMT tumor tissue was cut into 1-mm fragments using a scalpel and placed using fine forceps into the center of a 20-μl dome of alginate on top of a parafilm sheet. The domes were inverted into calcium chloride solution (102 mM calcium chloride and 15 mM Hepes) and allowed to polymerize for 10 min. Encapsulated tissue was then washed in DMEM, and fragments were cultured in 700 μl of DMEM/10% FBS in individual wells of a 48-well plate. Medium was changed every second day. To remove alginate before fixation, fragments were transferred to sodium citrate solution (0.2 M tri-sodium citrate and 0.1 M EDTA) for 5 min at room temperature (RT).

### Preparation of mammary gland whole mount specimens

Whole mounting of mammary fat pads was carried out as previously detailed (*101*). Intact inguinal glands were spread onto glass slides and fixed for 3 hours in Carnoy’s fixative (60% ethanol, 30% chloroform, and 10% glacial acetic acid) before washing in 70% ethanol for 15 min that was gradually changed to water. Glands were stained with Carmine alum (200 mg of Carmine dye and 500 mg of aluminum potassium in 100 ml H_2_O) overnight at 4°C, dehydrated through graduated ethanol, and then cleared in xylene. Glands were mounted using Permount (Thermo Fisher Scientific) and imaged using an Olympus dissecting microscope with the OpenLab software.

### Histology

Sections (4 μm) of FFPE tissues were immobilized on Superfrost Plus (Thermo Fisher Scientific) histological slides. Hematoxylin and eosin staining was carried out by dewaxing and rehydrating sections through xylene and graduated ethanol, followed by 5 min in Harris Hematoxylin (Sigma-Aldrich), rinsing in water purified by reverse osmosis (RO), three dips in 0.3% acid-alcohol (70% ethanol and 0.3% hydrochloric acid in H_2_O), 1 min in Scott’s Tap Water (10 g of sodium hydrogen carbonate and 10 g of magnesium sulfate in 5 liters of H_2_O), and 30 s in Eosin (Thermo Fisher Scientific). Stained sections were then dehydrated through graduated ethanol, cleared in xylene, and mounted in DPX (Sigma-Aldrich).

### Immunohistochemistry and immunofluorescence analysis of FFPE tissues

Immunofluorescence analyses on tissues were performed as previously described (*40*). Briefly, FFPE sections were dewaxed and rehydrated through xylene and graduated ethanol, and antigen retrieval was performed by boiling slides in a pressure cooker in citrate buffer [0.01 M citric acid in deionized water (pH 6.0)] or tris-EDTA buffer [10 mM tris-Cl and 1 mM EDTA in deionized water (pH 9.0)]. Labeling with antibodies was carried out in blocking buffer of 5% normal goat serum prepared in CAS-Block (Thermo Fisher Scientific). Full details of antibodies and dilutions are in the attached table S1. Slides were mounted with VECTASHIELD mounting medium (Vector Laboratories) or ProSciTech soft-set medium supplemented with 4′,6-diamidino-2-phenylindole (DAPI), and images were acquired using a Zeiss LSM 700 confocal system. ImageJ (National Institutes of Health) was used to calculate area covered by signal or integrated density per image after conversion to a binary image based upon a single manually determined threshold value applied across all images.

For immunohistochemical analyses, sections were prepared and labeled with primary antibody as above, with one exception that antigen retrieval for Ki67 was carried out in a decloaking chamber (Biocare Medical) at 95°C for 30 min. Details of antibodies and dilutions are in the attached table S1. Specific antibody binding was detected using EnVision Dual Link-HRP, followed by incubation with diaminobenzidine substrate (both from Dako). Sections were counterstained with Harris Hematoxylin and slides were mounted with DPX. Slides were scanned using a NanoZoomer Digital Pathology System (Hamamatsu Photonics). Positive pixels were quantified using Aperio ImageScope software (Leica Biosystems) using the programmed algorithm Positive Pixel Count v9.

### Immunofluorescence analysis of cells

Immunofluorescence analyses of cells grown on coverslips were performed as previously described (*102*). Cells were fixed in formalin (20 min at RT), quenched using 100 μM glycine in PBS, permeabilized with 20 μM glycine/0.1% saponin in PBS, and blocked with 10% FBS/0.1% saponin in PBS. Cells were labeled using antibodies detailed in the attached table S1. Coverslips were mounted with VECTASHIELD mounting medium supplemented with DAPI, and images were acquired using a Zeiss LSM 700 confocal system.

Immunofluorescence analyses of cells embedded within collagen were performed as previously described (*10*). Briefly, embedded cells were fixed in formalin, permeabilized with 0.2% Triton-X in PBS for 20 min with gentle shaking, and blocked with 0.2% bovine serum albumin (BSA) in PBS for at least 6 hours. Cells were labeled using antibodies detailed in the attached table S1. Alexa Fluor–conjugated phalloidin (Thermo Fisher Scientific) was used to label F-actin where indicated. For detection of BrdU incorporation in cells within collagen matrices, matrices were fixed in formalin for 30 min at RT, incubated for 20 min at RT in denaturing solution (3 N HCl/0.5% Tween 20), and washed for 5 min in PBS/0.5% BSA; and then, 0.1 M sodium borate (pH 8.5) was added for 2 min at RT to neutralize the acid. Matrices were washed as before, and mouse anti-BrdU primary antibody was added overnight in PBS/0.5% BSA at 4°C. Matrices were washed again before addition of secondary antibody for 1 hour at RT in the dark. DAPI (2 μg/ml in PBS) was added to all collagen matrices in PBS. Matrices were spread onto coverslips using forceps immediately before image acquisition. Images were acquired using a Zeiss LSM 700 confocal system. Where noted, confocal z-stacks were generated at 5-μm intervals. Resulting stacks were processed using Zeiss ZEN software to generate maximum projection orthogonal and depth-coded views and Fiji to plot z-profiles (the mean gray value per slice over the range of the z-stack after thresholding).

### Quantitative real-time PCR

Total RNA was prepared with TRIzol (Thermo Fisher Scientific) following the manufacturer’s instructions. As cells were embedded in collagen, whole collagen matrices were first homogenized in TRIzol using a FastPrep Homogenizer and Lysing Matrix A tubes (MP Bio) before transferring lysates to a fresh tube for subsequent RNA extraction. cDNA was prepared using the QuantiTect Reverse Transcription kit (QIAGEN), and quantitative real-time PCR was performed using the QuantiTect SYBR Green PCR Kit with a Rotor-Gene Q (both QIAGEN) following the manufacturers’ instructions. Results were normalized to glyceraldehyde-3-phosphate dehydrogenase (Gapdh) or 18*S*. Primers used were the following: Piezo1, Mm_Piezo1_3_SG QuantiTect Primer Assay QT01199142; Fn1/Fibronectin, Mm_Fn1_1_SG QuantiTect Primer Assay QT00135758; Col1a2/Collagen 1, Mm_Col1a2_2_SG QuantiTect Primer Assay QT02325736; Snai1/Snail, GAAGATGCACATCCGAAGC (forward) and ATGGCTTCTCACCAGTGTGG (reverse); Snai2/Slug, CTCACCTCGGGAGCATACAG (forward) and R-GACTTACACGCCCCAAGGATG (reverse); Zeb1, GCTGGCAAGACAACGTGAAAG (forward) and GCCTCAGGATAAATGACGGC (reverse); Zeb2, ATTGCACATCAGACTTTGAGGAA (forward) and ATAATGGCCGTGTCGCTTCG (reverse); Twist1, GGACAAGCTGAGCAAGATTCA (forward) and CGGAGAAGGCGTAGCTGAG (reverse); Twist2, CGCTACAGCAAGAAATCGAGC (forward) and GCTGAGCTTGTCAGAGGGG (reverse); 18S, GTAACCCGTTGAACCCCATT (forward) and CCATCCAATCGGTAGTAGCG (reverse); Gapdh, ACCCAGAAGACTGTGGATGG (forward) and CAGTGAGCTTCCCGTTCAG (reverse).

### Western analysis

Cells were lysed in radioimmunoprecipitation assay buffer [10 mM tris-Cl (pH 7.5), 5 mM EDTA, 1% (v/v) NP-40, 0.5% (w/v) sodium deoxycholate, 40 mM sodium tetrapyrophosphate, 1 mM sodium vanadate, 50 mM sodium fluoride, 1 mM phenylmethylsulfonyl fluoride, 0.025% (w/v) SDS, and 150 mM sodium chloride] containing protease and phosphatase inhibitors. Proteins were separated by 10 or 12% SDS–polyacrylamide gel electrophoresis in tris-glycine-SDS buffer and immobilized onto nitrocellulose membrane.

Western analysis was performed by blocking the membrane in 5% skimmed milk in tris-buffered saline/Tween 20 for 1 hour at RT, followed by addition of primary antibodies overnight at 4°C. Immunoblotting was carried out using antibodies detailed in table S1. Bands were visualized using a ChemiDoc Imaging System (Bio-Rad Laboratories Pty. Ltd.) or LAS 4000 (GE Healthcare) using either Pierce ECL or SuperSignal West Femto substrates (Thermo Fisher Scientific). Lane densities were quantified using ImageJ.

### Statistical analysis

The Prism (GraphPad) software was used for all analyses, except those carried out by KM Plotter. Please refer to relevant figure legends for *N* values, specific statistical tests used, and definitions of center and dispersion measures. For one-way analysis of variance (ANOVA) analyses, post hoc Tukey’s multiple comparisons test was used, and for Kruskal-Wallis analyses, post hoc Dunn’s multiple comparisons test was used. For unpaired parametric *t* tests, where variances were similar, Student’s was used, and when not, Welch’s correction was used. *P* values < 0.05 were considered statistically significant, with asterisks denoting significance level as *P < 0.05, **P < 0.01, ***P < 0.001, and ****P < 0.0001.
