# Supplementary material for: Compressive stress–driven Piezo1 activation and Rho-ROCK mechanotransduction promote tumor progression via epigenetic mechanical memory
Source: Sci Adv. 2026 Mar 4;12(10):eaeb1271. doi: 10.1126/sciadv.aeb1271 (PMC12959397; doi:10.1126/sciadv.aeb1271)
Supplement: Supplementary file 1 — Figs. S1 to S6 Table S1 [file sciadv.aeb1271_sm.pdf]

Science Advances
AAAS

Supplementary Materials for

Compressive stress–driven Piezo1 activation and Rho-ROCK mechanotransduction promote tumor progression via epigenetic mechanical memory

Sarah T. Boyle *et al.*

Corresponding author: Sarah T. Boyle, sarah.boyle@adelaide.edu.au;
Michael S. Samuel, michael.samuel@adelaide.edu.au

Sci. Adv. **12**, eaeb1271 (2026)
DOI: 10.1126/sciadv.aeb1271

This PDF file includes:

Figs. S1 to S6
Table S1

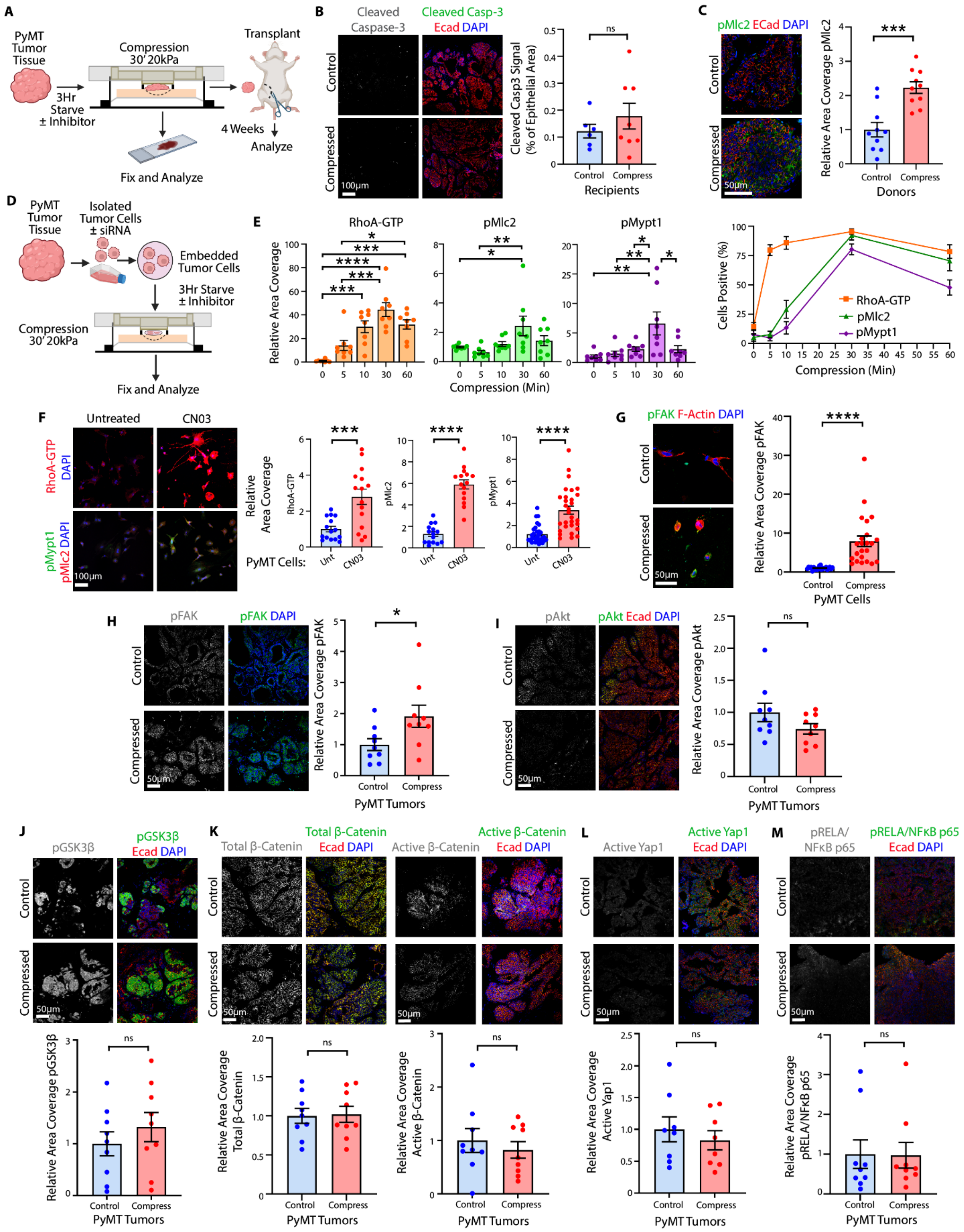

Fig. S1, Associated with Figure 1. A) Schematic illustrating the general process of tumor tissue compression for transplantation and analysis of signaling. **B)** Quantitative immunofluorescence of cleaved Caspase-3 (green) in tumor lesions formed from transplantation of PyMT tumor tissue

subjected to compressive stress. Samples are also labeled with E-cadherin (red) and DAPI (blue). Scale bar: 100 µm. Chart shows percent area coverage of signal in epithelial area as Mean±SEM (n=6 Control tumors, n=8 Compressed), analyzed by unpaired t-test. **C)** Immunofluorescence labelling of pMlc2 (green) in donor PyMT tumor tissue for transplants. Samples are also labelled with E-cadherin (epithelia, red) and DAPI (nuclei, blue). Scale bar: 50 µm. Chart shows relative area coverage of signal as Mean±SEM (n=10 tumor fragments/group), analyzed by unpaired t-test. ***p<0.001. **D)** Schematic illustrating the general process for compression of cells embedded in collagen matrices. **E)** PyMT tumor cells were embedded in collagen and subjected to compressive stress for times specified. Left: Relative area coverage of immunofluorescent signal of RhoA-GTP, p(Ser19)Mlc2 and p(Thr696)Mypt1, Mean±SEM (n=8 fields of view [FOV]/timepoint) analyzed by ANOVA. *p<0.05, **p<0.01, ***p<0.001, ****p<0.0001. Right: Proportion of cells positive for RhoA-GTP, pMlc2 and pMypt1, Mean±SEM (n=8 FOV/timepoint). **F)** Quantitative immunofluorescence analysis of RhoA-GTP, pMlc2 and pMypt1 (colors as specified) in PyMT tumor cells grown on coverslips and treated with CN03. Scale bar: 100 µm. Cells are also labelled with DAPI (blue). Data are Mean±SEM (n=3 coverslips, 5 FOV/coverslip RhoA-GTP/pMlc2; n=6 coverslips, 5 FOV/coverslip pMypt1), analyzed by unpaired t-tests. ***p<0.001, ****p<0.0001. **G)** Quantitative immunofluorescence of pFAK (green) in PyMT tumor cells embedded in collagen and subjected to compressive stress. Cells are also labeled for F-actin (red) and DAPI (blue). Scale bar: 50 µm. Chart shows relative area coverage of signal of 4 pooled experiments as Mean±SEM (n=22 FOV/group), analyzed by unpaired t-test. ****p<0.0001. **H)** Quantitative immunofluorescence of pFAK (greyscale and green) in PyMT tumor tissue subjected to compressive stress. Samples are also labelled with DAPI (blue). Scale bar: 50 µm. Chart shows relative area coverage of signal as Mean±SEM (n=9 tumors/group), analyzed by unpaired t-test. *p<0.05. **I)** Quantitative immunofluorescence of pAkt (greyscale and green) in PyMT tumor tissue subjected to compressive stress. Samples are also labeled with E-cadherin (red) and DAPI (blue). Scale bar: 50 µm. Chart shows relative area coverage of signal as Mean±SEM (n=9 tumors/group), analyzed by unpaired t-test. **J)** Quantitative immunofluorescence of pGSK3β (greyscale and green) in PyMT tumor tissue subjected to compressive stress. Samples are also labeled with E-cadherin (red) and DAPI (blue). Scale bar: 50 µm. Chart shows relative area coverage of signal as Mean±SEM (n=9 tumors/group), analyzed by unpaired t-test. **K)** Quantitative immunofluorescence of total β-catenin (left) and active β-catenin (right) (greyscale and green) in PyMT tumor tissue subjected to compressive stress. Samples are also labeled with E-cadherin (red) and DAPI (blue). Scale bar: 50 µm. Charts show relative area coverage of signals as Mean±SEM (n=9 tumors/group), analyzed by unpaired t-tests. **L)** Quantitative immunofluorescence of active Yap1 (greyscale and green) in PyMT tumor tissue subjected to compressive stress. Samples are also labeled with E-cadherin (red) and DAPI (blue). Scale bar: 50 µm. Chart shows relative area coverage of signal as Mean±SEM (n=9 tumors/group), analyzed by unpaired t-test. **M)** Quantitative immunofluorescence of pRELA/NFκB p65 (greyscale and green) in PyMT tumor tissue subjected to compressive stress. Samples are also labeled with E-cadherin (red) and DAPI (blue). Scale bar: 50 µm. Chart shows relative area coverage of signal as Mean±SEM (n=9 tumors/group), analyzed by unpaired t-test. Schematics in (A, D) were created in BioRender. Samuel, M. (2025) https://BioRender.com/koonmh0

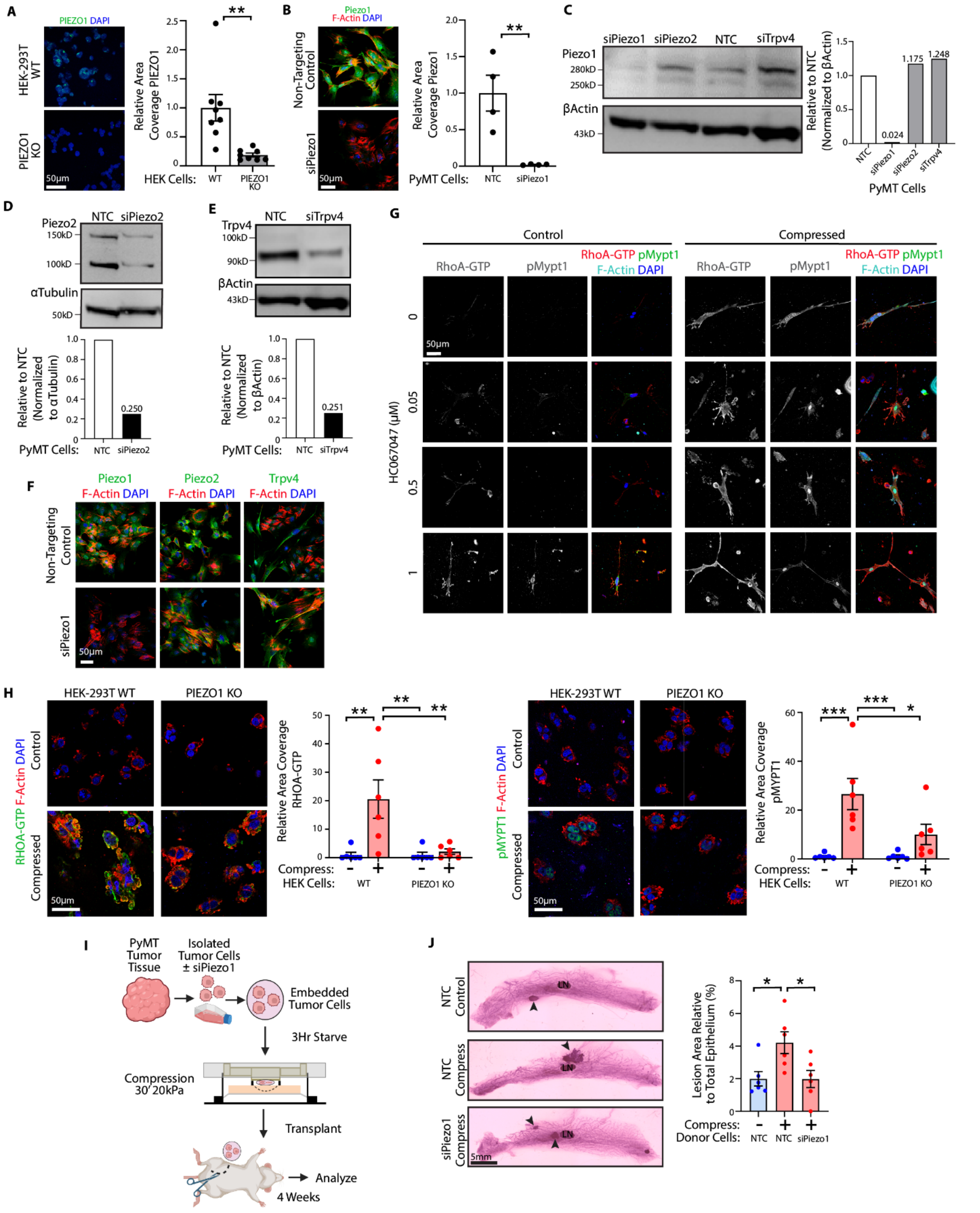

Fig. S2, Associated with Figure 2. A) Quantitative immunofluorescence of PIEZO1 (green) in wild-type and PIEZO1 CRISPR knockout HEK293T cells, fixed and encapsulated in agarose then embedded in paraffin for sectioning. Cells are also labeled with DAPI (blue). Scale bar: 50 µm.

Chart shows relative area coverage PIEZO1 as Mean±SEM (n=8 FOV/group), analyzed by unpaired t-test. **p<0.01. **B)** Quantitative immunofluorescence of Piezo1 (green) in PyMT tumor cells following transfection with NTC or siRNA targeting Piezo1. Cells are also labeled for F-actin (red) and DAPI (blue). Scale bar: 50 µm. Chart shows relative area coverage of Piezo1 as Mean±SEM (n=4 FOV/group), analyzed by unpaired t-test. **p<0.01. **C)** Western analysis of Piezo1 following transfection of primary PyMT tumor cells with NTC, or siRNA targeting Piezo1, Piezo2 or Trpv4. Chart shows mean densitometry of bands relative to control cells (normalized to loading). **D)** Western analysis of Piezo2 in PyMT tumor cells transfected with non-targeting control (NTC) siRNA or siRNA targeting Piezo2. Chart shows mean densitometry of bands relative to control cells (normalized to loading). **E)** Western analysis of Trpv4 following transfection of primary PyMT tumor cells with NTC or siRNA targeting Trpv4. Chart shows mean densitometry of bands relative to control cells (normalized to loading). For **C)** and **E)**, experiments were performed with a single non-targeting control group and blotted for either Piezo1 (C) or Trpv4 (E). **F)** Immunofluorescent labelling of Piezo1, Piezo2 or Trpv4 (green) in PyMT tumor cells following transfection with NTC or siRNA targeting Piezo1. Cells are also labeled for F-actin (red) and DAPI (blue). Scale bar: 50 µm. **G)** RhoA-GTP (greyscale and red) and pMypt1 (greyscale and green) in collagen-embedded PyMT tumor cells treated with Trpv4 inhibitor HC067047 at concentrations specified and subjected to compressive stress. Cells are also labeled for F-actin (cyan) and DAPI (blue). Scale bar: 50 µm. **H)** Quantitative immunofluorescence of RHOA-GTP and pMYPT1 (green) in wild-type and PIEZO1 CRISPR knockout HEK293T cells, embedded in collagen and subjected to compressive stress. Cells are also labeled for F-actin (red) and DAPI (blue). Scale bars: 50 µm. Charts show relative area coverage signal as Mean±SEM (n=6 FOV/group), analyzed by ANOVA tests. *p<0.05, **p<0.01, ***p<0.001. **I)** Schematic illustrating the process of transplanting cells embedded in collagen, following *Piezo1* knockdown and compression. **J)** Wholemounts of recipient glands that received transplantation of cells in which *Piezo1* was knocked down and compression applied. Scale bar: 5 mm. Chart shows the percent area of lesions/total epithelial area as Mean±SEM (n=6/group), analyzed by ANOVA. *p<0.05. Schematic in (I) was created in BioRender. Samuel, M. (2025) https://BioRender.com/koonmh0

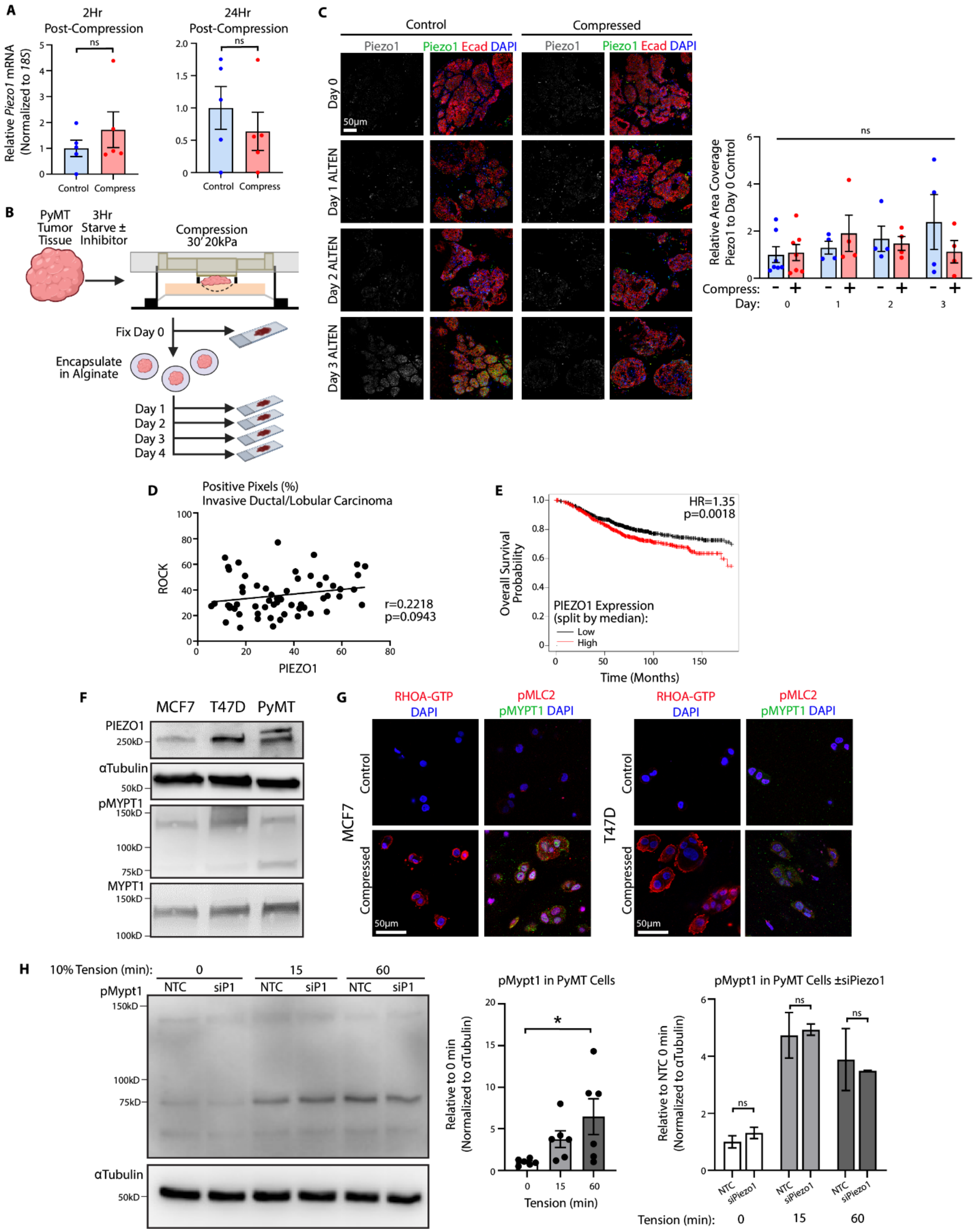

Fig. S3, Associated with Figure 2. A) mRNA levels of *Piezo1* in PyMT tumor cells subjected to compressive stress, after 2 hours and 24 hours as specified. Data are Mean±SEM (n=5 collagen matrices/group), analyzed by unpaired t-tests. **B)** Schematic illustrating the general process for

ALTEN culture to analyze tumor tissue following compression. **C)** Quantitative immunofluorescence of Piezo1 (greyscale and green) in PyMT tumor tissue cultured in ALTEN following compression. Samples are also labeled with E-cadherin (red) and DAPI (blue). Scale bar: 50 µm. Chart shows area coverage of signal relative to Day 0 control as Mean±SEM (n=7 tumor fragments/group Day 0, n=4/group Day 1-3), analyzed by unpaired t-tests (per Day) and ANOVA (entire time course). **D)** Correlation of PIEZO1 and ROCK positive pixel analyses of human breast cancer tissue microarrays in invasive ductal and lobular carcinoma (n=58 samples). Associations were compared using Spearman correlation coefficient, two-sided test. **E)** Overall survival based on *PIEZO1* expression in breast cancer patient samples from the TCGA database as analyzed using KM Plotter, patients split by median with follow-up threshold of 180 months. n=940 low expression, 939 high expression. **F)** Western analysis of PIEZO1 and pMYPT1 levels in human breast cancer cell lines MCF7 and T47D. **G)** Immunofluorescence of RHOA-GTP, pMLC2 and pMYPT1 (colors as specified) in MCF7 and T47D human breast cancer cells, embedded in collagen and subjected to compressive stress. Cells are also labeled with DAPI (blue). Scale bars: 50 µm. **H)** Western analysis of phosphorylated Mypt1 in primary PyMT tumor cells transfected with NTC or siRNA targeting Piezo1, and subjected to static tensile whole-cell stretch at 10% elongation for times specified. Left chart shows relative densitometry of NTC sample bands (normalized to loading) as Mean±SEM from 3 pooled experiments (n=6/condition), analyzed by ANOVA, *p<0.05. Right chart shows representative relative densitometry of NTC and siPiezo1 sample bands (normalized to loading) as Mean±SEM (n=2/condition), analyzed by ANOVA. Schematic in (B) was created in BioRender. Samuel, M. (2025) https://BioRender.com/koonmh0

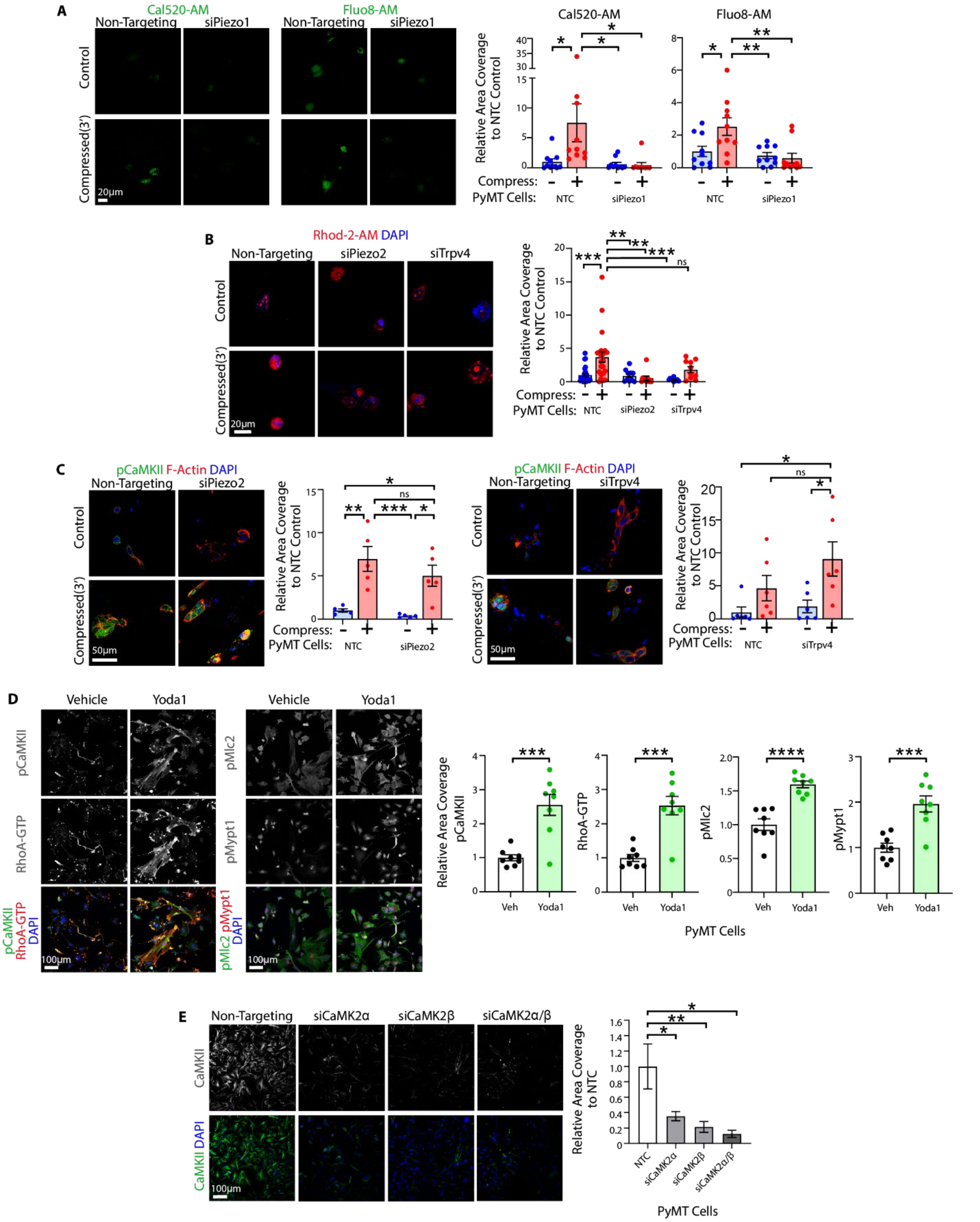

Fig. S4, Associated with Figure 3. A) Cal520-AM and Fluo8-AM fluorescence (green) in PyMT tumor cells with and without *Piezo1* knockdown, embedded in collagen and subjected to compressive stress for 3 minutes. Scale bar: 20 µm. Charts show relative area coverage of signals as Mean±SEM (n=10 FOV/group), analyzed by ANOVA. *p<0.05, **p<0.01. **B)** Rhod-2-AM

fluorescence (red) in PyMT tumor cells following *Piezo2* or *Trpv4* knockdown, embedded in collagen and subjected to compressive stress for 3 minutes. Nuclei are labelled with DAPI (blue). Scale bar: 20 µm. Chart shows relative area coverage of signal as Mean±SEM of 2 pooled experiments (n=26 FOV NTC Control, 22 FOV NTC Compressed, 10 FOV siPiezo2/Trpv4 Control/Compressed), analyzed by ANOVA. **p<0.001, ***p<0.001. **C)** Quantitative immunofluorescence of pCaMKII (green) in PyMT tumor cells with and without *Piezo2* (left) or *Trpv4* (right) knockdown, embedded in collagen and subjected to compressive stress for 3 minutes. Cells are also labeled for F-actin (red) and DAPI (blue). Scale bars: 50 µm. Charts show relative area coverage as Mean±SEM (n=5 FOV/group Piezo2, 6 Trpv4), analyzed by ANOVA. *p<0.05, **p<0.01, ***p<0.001. **D)** Quantitative immunofluorescence analysis of pCaMKII, RhoA-GTP, pMlc2 and pMypt1 (colors as specified) in PyMT tumor cells grown on coverslips and stimulated with Yoda1. Scale bars: 100 µm. Cells are also labelled with DAPI (blue). Data are Mean±SEM (n=8 FOV/condition), analyzed by unpaired t-tests. ***p<0.001, ****p<0.0001. **E)** Quantitative immunofluorescence analysis of CaMKII (greyscale and green) in PyMT tumor cells grown on coverslips, transfected with NTC siRNA or siRNA targeting CaMKIIα, CaMKIIβ or both. Scale bar: 100 µm. Cells are also labelled with DAPI (blue). Data are Mean±SEM of 2 pooled experiments (n=10 FOV NTC/individual siRNA, 6 dual-knockdown), analyzed by ANOVA. *p<0.05, **p<0.01.

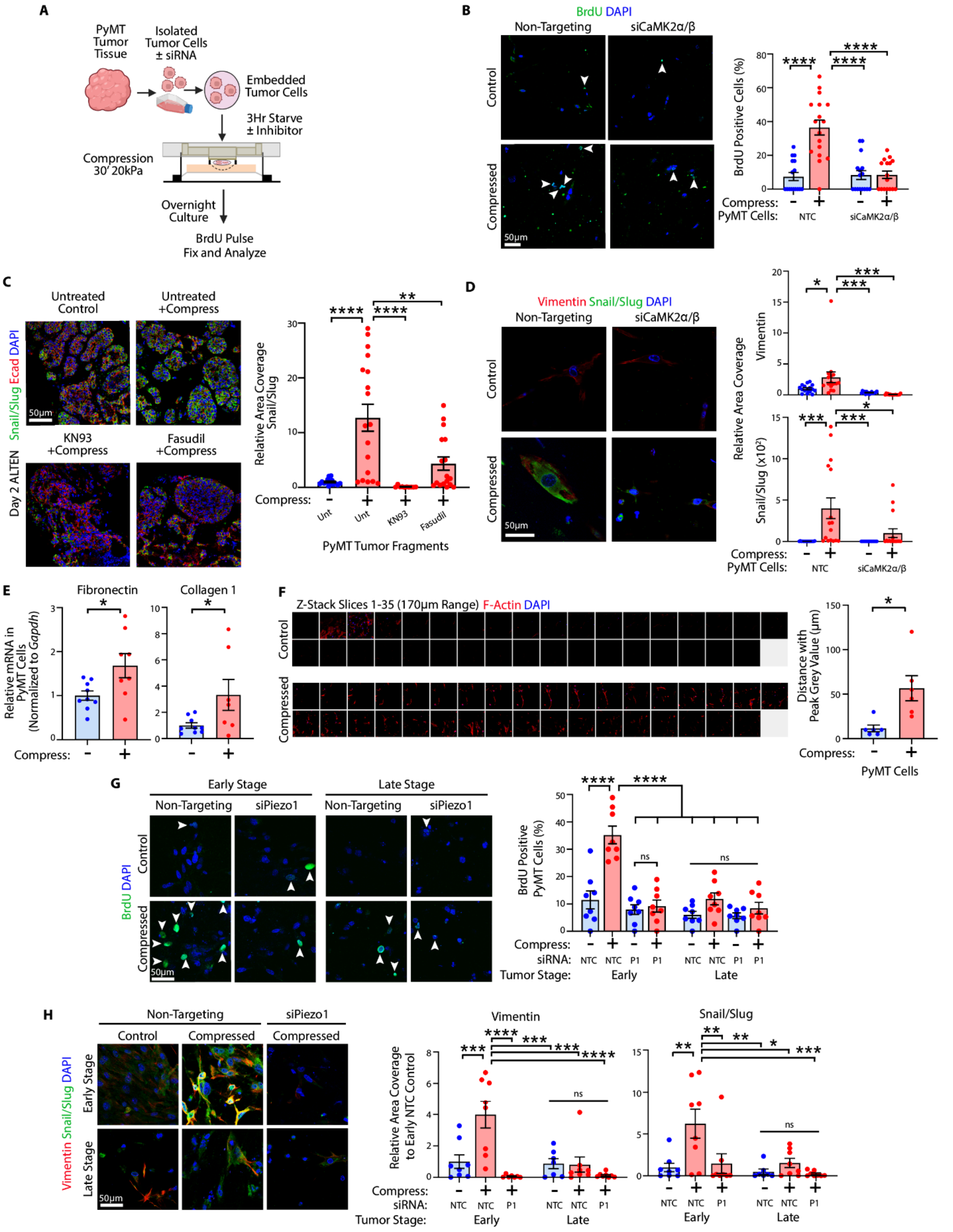

Fig. S5, Associated with Figures 4 and 5. A) Schematic illustrating the process of assessing cells in collagen following compression and overnight culture. **B)** BrdU incorporation (green) in PyMT

tumor cells transfected with non-targeting control siRNA or siRNA targeting CaMKIIα and CaMKIIβ, embedded in collagen and subjected to compressive stress. Cells are also labeled with DAPI (blue). Scale bar: 50μm. Chart shows percent BrdU-positive cells as Mean±SEM of 2 pooled experiments (n=16 FOV NTC Control/siCaMKII, 17 NTC Compressed), analyzed by ANOVA. ****p<0.0001. **C)** Quantitative immunofluorescence of Snail/Slug (green) in PyMT tumor tissue pre-treated with KN93 or Fasudil, subjected to compressive stress and cultured in ALTEN. Samples are also labeled for E-cadherin (red) and DAPI (blue). Scale bar: 50 μm. Chart shows relative area coverage of signal as Mean±SEM from 2 pooled experiments (n=15 FOV Control, 18 Compressed, 10 KN93, 17 Fasudil), analyzed by ANOVA. **p<0.01, ****p<0.0001. **D)** Quantitative immunofluorescence of Vimentin (red) and Snail/Slug (green) in PyMT tumor cells transfected with non-targeting control siRNA or siRNA targeting CaMKIIα and CaMKIIβ, embedded in collagen, subjected to compressive stress and cultured overnight. Nuclei are labelled with DAPI (blue). Scale bars: 50 μm. Charts show relative area coverage of signal per cell as Mean±SEM of 2 pooled experiments (n=16 FOV/group), analyzed by ANOVA. *p<0.05, ***p<0.001. **E)** mRNA levels of *Fn1* and *Col1a2* in PyMT tumor cells embedded in collagen matrices and subjected to compressive stress. Data are Mean±SEM from 3 pooled experiments (Fibronectin: n=9 Control, 8 Compressed; Collagen 1: n=9 Control, 7 Compressed), analyzed by unpaired t-tests. *p<0.05. **F)** Representative Z-stack slices of collagen containing tumor cells, compressed and cultured for 4 days. Cells were labelled with phalloidin (F-actin, red) and DAPI (blue). Chart shows the distance exhibiting peak grey value as Mean±SEM (n=6 FOV/group), analyzed by unpaired t-test. *p<0.05. **G)** BrdU incorporation (green) in either early stage (7-8 weeks old) or late stage (10 weeks old) PyMT tumor cells with and without *Piezo1* knockdown, embedded in collagen and subjected to compressive stress. Cells are also labeled with DAPI (blue). Scale bar: 50μm. Chart shows percent BrdU-positive cells as Mean±SEM (n=8 FOV/group), analyzed by ANOVA. ****p<0.0001. **H)** Quantitative immunofluorescence of Vimentin (red) and Snail/Slug (green) in either early stage (7-8 weeks old) or late stage (10 weeks old) PyMT tumor cells with and without *Piezo1* knockdown, embedded in collagen, subjected to compressive stress. Nuclei are labelled with DAPI (blue). Scale bars: 50 μm. Charts show relative area coverage of signal per cell as Mean±SEM (Vimentin: n=8 FOV Early NTC Control/Compressed and Late NTC/siPiezo1 Compressed, 7 Early siPiezo1 Compressed and Late NTC Control; Snail/Slug: n=8 FOV Early NTC/siPiezo1 Control/Compressed and Late NTC/siPiezo1 Compressed, 7 Late NTC Control), analyzed by ANOVA tests. *p<0.05, **p<0.01, ***p<0.001, ****p<0.0001. Schematic in (A) was created in BioRender. Samuel, M. (2025) https://BioRender.com/koonmh0

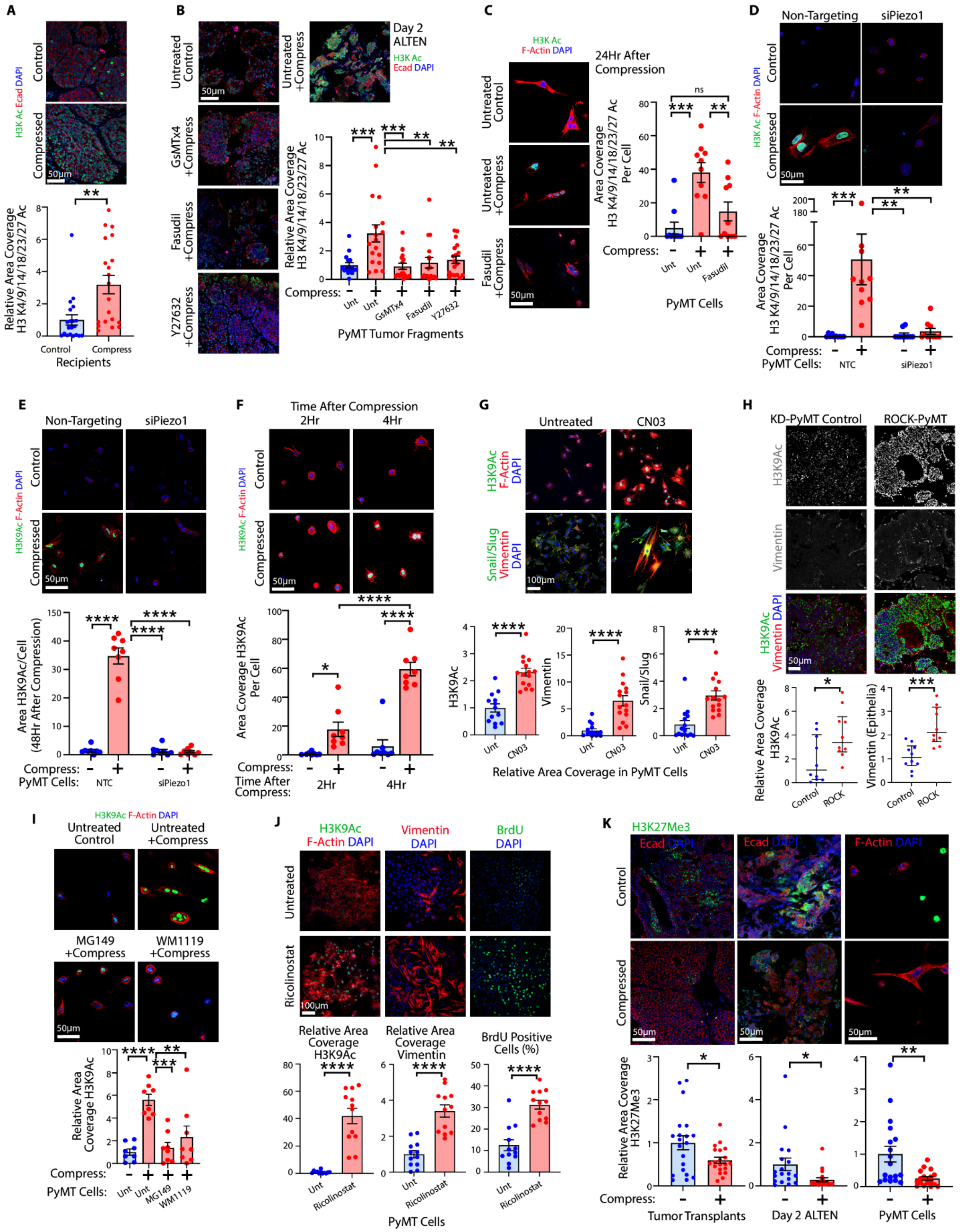

Fig. S6, Associated with Figure 6. A) Quantitative immunofluorescence of histone H3 acetylation on lysine residues K4, 9, 14, 18, 23 and 27 (H3K Ac, green) in tumor lesions formed from transplantation of PyMT tumor tissue subjected to compressive stress. Samples are also labeled

with E-cadherin (red) and DAPI (blue). Scale bar: 50 µm. Chart shows relative area coverage of signal as Mean±SEM of 3 pooled experiments (n=20 tumors/group), analyzed by unpaired t-test. **p<0.01. **B)** Quantitative immunofluorescence of H3K4/9/14/18/23/27 Ac (green) in PyMT tumor tissue pre-treated with GsMTx4, Fasudil or Y27632, subjected to compressive stress and cultured in ALTEN. Samples are also labeled with E-cadherin (red) and DAPI (blue). Scale bar: 50 µm. Chart shows relative area coverage of signal as Mean±SEM from 2 pooled experiments (n=16 FOV Control, 18 Compressed/GsMTx4/Y27632, 17 Fasudil), analyzed by ANOVA. **p<0.01, ***p<0.001. **C)** Quantitative immunofluorescence of H3K4/9/14/18/23/27 Ac (green) in PyMT tumor cells embedded in collagen, treated with Fasudil, subjected to compressive stress and cultured overnight. Cells are also labeled for F-actin (red) and DAPI (blue). Scale bar: 50 µm. Chart shows area coverage of signal per cell as Mean±SEM (n=10 FOV/group), analyzed by ANOVA. **p<0.01, ***p<0.001. **D)** Quantitative immunofluorescence of H3K4/9/14/18/23/27 Ac (green) in PyMT tumor cells with and without *Piezo1* knockdown, embedded in collagen, subjected to compressive stress and cultured overnight. Cells are also labeled for F-actin (red) and DAPI (blue). Scale bar: 50 µm. Chart shows area coverage of signal per cell as Mean±SEM (n=10 FOV/group), analyzed by ANOVA. **p<0.01, ***p<0.001. **E)** Quantitative immunofluorescence of H3K9Ac (green) in PyMT tumor cells with and without *Piezo1* knockdown, embedded in collagen, subjected to compressive stress and cultured for 48 hours. Cells are also labeled for F-actin (red) and DAPI (blue). Scale bar: 50 µm. Chart shows area coverage of signal per cell number as Mean±SEM (n=8 FOV/group), analyzed by ANOVA. ****p<0.0001. **F)** Quantitative immunofluorescence of H3K9Ac (green) in PyMT tumor cells embedded in collagen, subjected to compressive stress and cultured a further 2 or 4 hours. Cells are also labeled for F-actin (red) and DAPI (blue). Scale bar: 50 µm. Chart shows area coverage of signal per cell as Mean±SEM (n=8 FOV/group), analyzed by ANOVA. *p>0.05, ****p<0.0001. **G)** Quantitative immunofluorescence analysis of H3K9Ac, Snail/Slug and Vimentin (colors as specified) in PyMT tumor cells grown on coverslips, 24 hours post-treatment with CN03. Scale bar: 100 µm. Cells are also labelled with F-Actin and DAPI as specified. Data are Mean±SEM of 3 coverslips (H3K9Ac: n=13 FOV untreated, 15 CN03; Vim and Snail/Slug: n=15 FOV/condition), analyzed by unpaired t-tests.****p<0.0001. **H)** Quantitative immunofluorescence of H3K9Ac (greyscale and green) and Vimentin (greyscale and red) in PyMT tumors in which ROCK has been activated (ROCK-PyMT) compared to kinase-dead controls (KD-PyMT Control) with endogenous levels of ROCK activity. Samples are also labelled with DAPI (blue). Scale bar: 50 µm. Chart shows relative area coverage of signals as Median±IQR (n=10 tumors/group), analyzed by Mann-Whitney test. *p<0.05, ***p<0.001. **I)** Quantitative immunofluorescence of H3K9Ac (green) in PyMT tumor cells embedded in collagen, treated with inhibitors of Tip60 (MG149) or KAT6A (WM1119), subjected to compressive stress and cultured overnight. Cells are also labeled for F-actin (red) and DAPI (blue). Scale bar: 50 µm. Chart shows relative area coverage of H3K9Ac as Mean±SEM (n=8 FOV/group), analyzed by ANOVA. **p<0.01, ***p<0.001, ****p<0.0001. **J)** Quantitative immunofluorescence analysis of H3K9Ac (green) and Vimentin (red), and BrdU incorporation (green), in PyMT tumor cells grown on coverslips and treated with HDAC6 inhibitor Ricolinostat. Scale bar: 100 µm. Cells are also labelled with F-Actin and DAPI as specified. Data are Mean±SEM of 2 coverslips (n=6 FOV/condition), analyzed by unpaired t-tests.****p<0.0001. **K)** Quantitative immunofluorescence of histone H3 trimethylation on lysine residue K27 (H3K27Me3, green) in; Left: tumor lesions formed from transplantation of PyMT tumor tissue subjected to compressive stress; Middle: PyMT tumor tissue subjected to compressive stress and cultured in ALTEN; Right: PyMT tumor cells embedded in collagen, subjected to compressive

stress and cultured overnight. Tumor tissue samples are also labelled with E-cadherin (red) and DAPI (blue), cells for F-actin (red) and DAPI (blue). Scale bars: 50 µm. Charts show relative area coverage of signal as Mean±SEM of; Left: 3 pooled experiments (n=20 tumors/group), analyzed by unpaired t-test, *p<0.05; Middle: 2 pooled experiments (n=18 FOV Control, 19 Compressed), analyzed by unpaired t-test, *p<0.05; Right: 2 pooled experiments (n=18 FOV/group), analyzed by unpaired t-test, **p<0.01.

Table S1.
Antibodies used in immunofluorescence, immunohistochemistry and Western analyses.

Antibody	Species	Clone	Supplier	Cat #	Antigen Retrieval	Dilution	Incubation Temp (°C) [Time (hr)]
β-Actin	Rabbit	SP124	Abcam	ab115777	—	1:1000 (WB)	4[18] (WB)
p(Ser473)-Akt	Rabbit	736E11	Cell Signaling Technology	3787	10 mM Tris-Cl; 1 mM EDTA pH 8[a]	1:50 (IF FFPE)	4[18] (IF FFPE)
BrdU	Mouse	B44	BD Biosciences	347580	10 mM Tris-Cl; 1 mM EDTA pH 9[a]	1:100 (IF FFPE) 1:200 (IF Cells)	4[18] (IF FFPE, IF Cells)
E-Cadherin (pure/ AlexaFluor -555 conj/ AlexaFluor -647 conj)	Mouse	36	BD Biosciences	610182/ 560064/ 560062	10 mM Citrate pH 6[a] *OR* 10 mM Tris-Cl; 1 mM EDTA pH 8/9[a]	1:200 (IF FFPE)	4[18] (IF FFPE) *OR* 25[1] (IF FFPE, conjugated only)
CaMKII	Rabbit	EPR6686(2)	Abcam	ab134041	—	1:100 (IF Cells)	4[18] (IF Cells)
p(Thr286)-CaMKII	Rabbit	Polyclonal	Abcam	ab32678	10 mM Citrate pH 6[a] *OR* 10 mM Tris-Cl; 1 mM EDTA pH 9[a]	1:200 (IF FFPE, IF Cells)	4[18] (IF FFPE, IF Cells)
Total β-Catenin	Mouse	14	BD Biosciences	610154	10 mM Tris-Cl; 1 mM EDTA pH 8[a]	1:50 (IF FFPE)	4[18] (IF FFPE)
Active β-Catenin	Mouse	8.00E+07	Merck	05-665	10 mM Tris-Cl; 1 mM EDTA pH 9[a]	1:100 (IF FFPE)	4[18] (IF FFPE)

Cleaved Caspase-3	Rabbit	5A1E	Cell Signaling Technology	9664	10 mM Tris-Cl; 1 mM EDTA pH 9[a]	1:100 (IF FFPE)	4[18] (IF FFPE)
p(Tyr397)-FAK	Rabbit	141-9	Thermofisher Scientific	44-625G	10 mM Tris-Cl; 1 mM EDTA pH 9[a]	1:100 (IF FFPE)	4[18] (IF FFPE)
p(Ser9)-GSK3β	Rabbit	Polyclonal	Cell Signaling Technology	9336	10 mM Tris-Cl; 1 mM EDTA pH 9[a]	1:100 (IF FFPE)	4[18] (IF FFPE)
Histone H3 (acetyl K4+K9+K14+K18+K23+K27)	Rabbit	RM1045	Abcam	ab300641	10 mM Tris-Cl; 1 mM EDTA pH 9[a]	1:2000 (IF FFPE) 1:100 (IF Cells)	4[18] (IF FFPE, IF Cells)
Histone H3 (acetyl K9)	Rabbit	Y28	Abcam	ab32129	10 mM Tris-Cl; 1 mM EDTA pH 9[a]	1:500 (IF FFPE) 1:250 (IF Cells)	4[18] (IF FFPE, IF Cells)
Histone H3 (tri methyl K27)	Rabbit	EPR18607	Abcam	ab192985	10 mM Tris-Cl; 1 mM EDTA pH 9[a]	1:100 (IF FFPE) 1:1000 (IF Cells)	4[18] (IF FFPE, IF Cells)
Ki67	Mouse	K2	Leica Microsystems	KI67MM1LCE	10 mM Citrate pH 6[b]	1:100 (IHC FFPE)	4[18] (IHC FFPE)
p(Ser19)-Mlc2	Rabbit	Polyclonal	Cell Signaling Technology	3671	10 mM Tris-Cl; 1 mM EDTA pH 9[a]	1:50 (IF FFPE)	4[18] (IF FFPE)
p(Ser19)-Mlc2	Mouse	Monoclonal	Cell Signaling Technology	3675	—	1:50 (IF Cells)	4[18] (IF Cells)
MMP9	Rabbit	Polyclonal	Abcam	ab38898	10 mM Tris-Cl; 1 mM EDTA pH 9[a]	1:100 (IF FFPE)	4[18] (IF FFPE)

p(Thr696)-Mypt1	Rabbit	Polyclonal	Merck	ABS45	10 mM Citrate pH 6[a] *OR* 10 mM Tris-Cl; 1 mM EDTA pH 9[a]	1:100 (IF FFPE, IF Cells)	4[18] (IF FFPE, IF Cells)
Piezo1	Rabbit	Polyclonal	Abcam	ab128245	10 mM Tris-Cl; 1 mM EDTA pH 9[a]	1:100 (IF Cells, IF FFPE, IHC FFPE) 1:1000 (WB)	4[18] (IF FFPE, IHC FFPE, WB)
Piezo2	Rabbit	Polyclonal	Novus Biologicals	NBP1-78624	—	1:1000 (WB)	4[18] (WB)
Piezo2	Rabbit	Polyclonal	Abcam	ab243416	—	1:100 (IF Cells)	4[18] (IF Cells)
p(Ser536)-RELA/NFκB p65	Mouse	Monoclonal	Santa Cruz Biotechnology	sc-136548	10 mM Tris-Cl; 1 mM EDTA pH 9[a]	1:50 (IF FFPE)	4[18] (IF FFPE)
RhoA-GTP	Mouse	Monoclonal	New East Biosciences	26904	—	1:100 (IF Cells)	4[18] (IF Cells)
ROCK (1/2)	Rabbit	Polyclonal	Merck	07-1458	10 mM Citrate pH 6[a]	1:100 (IHC FFPE)	4[18] (IHC)
Snail/Slug	Rabbit	Polyclonal	Abcam	ab180714	10 mM Tris-Cl; 1 mM EDTA pH 9[a]	1:100 (IF FFPE, IF Cells)	4[18] (IF FFPE, IF Cells)
TRPV4	Rabbit	Polyclonal	Abcam	ab94868	—	1:1000 (WB) 1:100 (IF Cells)	4[18] (WB, IF Cells)
α-Tubulin	Mouse	DM1A	Abcam	ab7291	—	1:1000 (WB)	4[18] (WB)
Active YAP1	Rabbit	EPR19812	Abcam	ab205270	10 mM Tris-Cl; 1 mM	1:500 (IF FFPE)	4[18] (IF FFPE)

					EDTA pH 9[a]		
Vimentin	Mouse	LN-6	Sigma-Aldrich	V2258	10 mM Tris-Cl; 1 mM EDTA pH 9[a]	1:100 (IF FFPE, IF Cells)	4[18] (IF FFPE, IF Cells)
Phalloidin-AlexaFluor 594	—	—	Thermofisher Scientific	A12381	—	1:250 (IF Cells)	25[1] (IF Cells)
Phalloidin-AlexaFluor 647	—	—	Abcam	ab176759	—	1:1000 (IF Cells)	25[1] (IF Cells)
AlexaFluor 2° Abs	Goat/ Donkey	—	Thermofisher Scientific	Various	—	1:400 (IF FFPE, IF Cells)	25[1] (IF FFPE, IF Cells)
Horseradish Peroxidase-conjugated 2° Abs	Goat/ Donkey	—	Rockland Inc./ Abcam	Various	—	1:10,000 (WB, ECL Detection) 1:100,000 (WB, Femto Detection)	25[1] (WB)

[a]Antigen retrieval was carried out in a pressure cooker at 63 kPa above atmospheric pressure, with boiling for 15 minutes.
[b]Antigen retrieval was carried out in a decloaking chamber (Biocare Medical) at 95°C for 30 minutes.
Experimental conditions for immunofluorescence, immunohistochemistry and Western analyses. IF: Immunofluorescence; IHC: Immunohistochemistry; WB: Western Analysis; FFPE: Formalin-fixed paraffin-embedded sections.
